# Identification of metabolic markers predictive of prediabetes in a Korean population

**DOI:** 10.1038/s41598-020-78961-4

**Published:** 2020-12-15

**Authors:** Heun-Sik Lee, Tae-Joon Park, Jeong-Min Kim, Jun Ho Yun, Ho-Yeong Yu, Yeon-Jung Kim, Bong-Jo Kim

**Affiliations:** Division of Genome Research, Center for Genome Science, National Institute of Health, Osong Health Technology Administration Complex, 187, Osongsaengmyeong 2-ro, Osong-eup, Heungdeok-gu, Cheongju-si, Chungchenogbuk-do 28159 Republic of Korea

**Keywords:** Biochemistry, Molecular biology, Biomarkers

## Abstract

Prediabetes (PD) is a high-risk state of developing type 2 diabetes, and cardiovascular and metabolic diseases. Metabolomics-based biomarker studies can provide advanced opportunities for prediction of PD over the conventional methods. Here, we aimed to identify metabolic markers and verify their abilities to predict PD, as compared to the performance of the traditional clinical risk factor (CRF) and previously reported metabolites in other population-based studies. Targeted metabolites quantification was performed in 1723 participants in the Korea Association REsource (KARE) cohort, from which 500 normal individuals were followed up for 6 years. We selected 12 significant metabolic markers, including five amino acids, four glycerophospholipids, two sphingolipids, and one acylcarnitine, at baseline, resulting in a predicted incidence of PD with an area under the curve (AUC) of 0.71 during follow-up. The performance of these metabolic markers compared to that of fasting glucose was significantly higher in obese patients (body mass index: BMI ≥ 25 kg/m^2^, 0.79 vs. 0.58, *P* < 0.001). The combination with metabolic markers, CRF, and fasting glucose yielded the best prediction performance (AUC = 0.86). Our results revealed that metabolic markers were not only associated with the risk of PD, but also improved the prediction performance in combination with conventional approaches.

## Introduction

Prediabetes (PD) is an intermediate state between normal glucose tolerance (NGT) and overt type 2 diabetes (T2D). T2D is a multifactorial chronic disease that results from combined environmental and genetic risk factors that affect insulin resistance (IR) and impaired glucose tolerance (IGT)^1^. Approximately 37% and 70% of individuals with PD may be at a high-risk of developing T2D or related complications within 4 and 10 years, respectively^2,3^. The prevalence of T2D can be reduced, delayed, or even prevented by early screening for PD and effective interventions such as dietary changes, increased physical activity, and clinical treatment^4^. Similarly to T2D, PD diagnosis is based on biochemical criteria, such as impaired fasting glucose (IFG), IGT, combined IFG/IGT, glycated hemoglobin (HbA1c), or IR, which are early indicators of progression to diabetes^5^. Additionally, several traditional clinical risk factors (CRF), such as age, sex, body mass index (BMI), high-density lipoprotein (HDL) and low-density lipoprotein (LDL) cholesterol (HDL-C, LDL-C), and triglyceride (TG) can predict future diabetes risk. However, detecting PD using these indicators is tedious and time-consuming, as well as prone to inconsistencies in a condition-dependent manner in patients^6,7^. Furthermore, they have moderate or low sensitivity in PD diagnosis and are typically examined after years of subclinical metabolic changes^8,9^. Therefore, there has been an increasing interest in developing predictive methods by identifying molecular biomarkers to improve the understanding of specific biochemical changes preceding PD onset. These studies would allow the patients to adopt preventive lifestyle actions or successful clinical interventions based on monitoring of the molecular markers and may lead to the development of effective medicines.

Metabolomics is a high-throughput technology for identifying endogenous metabolic markers with diverse biochemical properties to provide insights into the pathogenesis of diseases and diagnostic approaches^10,11^. In the last decade, several studies have evaluated the associations between a wide range of metabolites and PD using targeted or non-targeted metabolomics approaches^12,13^. To date, branched-chain amino acids (BCAAs) (isoleucine, leucine, and valine), other amino acids (glycine, glutamine, glutamate, and aromatic amino acids), sugars (glucose and fructose), and lipid subclasses (phospholipids, sphingomyelins, triglycerides, palmitate, and palmitoleate) have been associated with the progression of PD^12,14,15^. However, because these metabolites were reported mostly in Caucasian and European population-based cohort studies, further studies of different racial and ethnic groups, such as Asian populations, are needed. A recent metabolomics study reported PD-related metabolites, including methylcysteine and sedoheptulose 1,7-bis-phosphate in a Japanese population with IFG^16^. The results obtained using a non-targeted metabolomics approach revealed a set of five metabolites, including 20-hydroxy-leukotriene E4, lysophosphatidylcholine (lysoPC) (C20:3, C20:4), 5-methoxytryptamine and endomorphin-1, in a Chinese population with IFG and IGT^17^. Another targeted metabolomics approach identified 7 metabolites, including BCAAs and lysoPC (C16:0, C18:0, C18:1, C18:2), in a Chinese population with IFG and T2D^18^. Therefore, in the present study, we identified novel metabolic markers for PD, estimated their predictive performance in a Korean population using a comprehensive targeted metabolomics approach, and compared them to other well-known predictors, including two previously reported models^12,15^, CRF, and fasting glucose levels.

## Results

### Subject characteristics

Table 1 shows the baseline characteristics of the participants, stratified by prevalent cases at baseline and incident cases in the follow-up. Compared to the participants who did not develop PD (NGT, n = 924), those with PD (n = 799) were older and had higher levels of BMI, LDL-C, TG, HbA1c, fasting glucose, 2 h-PPG, fasting insulin, and HOMA-IR, and lower levels of HDL-C, and were mostly female. The participants with incident PD (n = 199) had higher levels of TG, HbA1c, fasting glucose, 2 h-PPG, fasting insulin, and HOMA-IR compared to those without PD (n = 301) during the follow-up. Because PD can be observed form the dysglycemia and IR states, all participants at baseline were also reclassified according to IFG, IGT, combined IFG/IGT, HbA1c, and HOMA-IR levels (study characteristics are shown in Supplementary Table S1). According to the dysglycemic state, the sample set included 997, 255, 265, and 206 participants with NGT, IFG, IGT, and combined IFG/IGT (Supplementary Table S1A). According to HbA1c levels, the population corresponded to 1105 participants with NGT (HbA1c < 5.7) and 618 with PD (5.7 ≤ HbA1c ≤ 6.4) (Supplementary Table S1B). According to HOMA-IR levels, the sample set was divided into four quartiles: 431 in the 25th percentile (Q1), 431 in the 50th percentile (Q2), 430 in the 75th percentile (Q3), and 431 in the 100th percentile (Q4) of distribution (Supplementary Table S1C).

**Table 1:** Characteristics of the study populations in the baseline (KARE S2) and follow-up (KARE S5) cohorts by ADA criteria.

Clinical and laboratory parameters	Baseline (n = 1723)		Follow-up (n = 500)	
	NGT (n = 924)	PD (n = 799)	NGT (n = 301)	PD (n = 199)
Age (years)	54.54 ± 8.66	56.85 ± 8.70^*^	59.42 ± 7.71	62.07 ± 9.08^*^
Sex (female) (%)	50.11	58.46^*^	50.83	54.27^*^
BMI (kg/m^2^)	23.31 ± 2.91	25.29 ± 3.15^*^	22.98 ± 2.75	23.63 ± 3.37
HDL cholesterol (mg/dL)	45.46 ± 10.28	43.38 ± 10.13^*^	49.91 ± 13.06	48.03 ± 12.28
LDL cholesterol (mg/dL)	118.57 ± 29.74	124.42 ± 32.58^*^	115.66 ± 26.63	118.34 ± 30.82
Triglycerides (TG) (mg/dL)	110.74 ± 56.79	145.44 ± 69.85^*^	108.06 ± 50.25	124.95 ± 64.72^*^
HbA1c (%)	5.26 ± 0.29	5.74 ± 0.33^*^	5.16 ± 0.23	5.68 ± 0.29^*^
Fasting glucose (mg/dL)	82.91 ± 4.68	99.65 ± 8.74^*^	83.47 ± 5.44	91.70 ± 8.20^*^
2 h-PPG (mg/dL)	88.96 ± 15.94	143.20 ± 32.33^*^	89.74 ± 19.09	126.11 ± 34.01^*^
Fasting insulin (μU/mL)	6.70 ± 3.05	8.57 ± 4.36^*^	6.24 ± 2.20	8.04 ± 4.04^*^
HOMA-IR	1.38 ± 0.67	2.12 ± 1.14^*^	1.29 ± 0.49	1.85 ± 1.05^*^

Data presented as mean ± deviations (SD) and n (%). *BMI* body mass index, *HDL* high density lipoprotein, *LDL* low-density lipoprotein, *HbA1c* glycated hemoglobin. *2 h-PPG* 2 h-postprandial glucose. HOMA-IR (homeostasis model assessment of insulin resistance) was calculated by following formula = [Fasting insulin (µlU/mL) × Fasting glucose (mg/dL)]/405. *Mann–Whitney test analyzed the significance between the two groups (*P* < 0.05).

### Significantly changed metabolites in PD and PD-related biochemical traits

To identify significant metabolites with altered serum concentrations between individuals with NGT and PD, as well as PD-related traits, we performed multivariable logistic regression analysis based on pairwise comparisons (PD vs. NGT by the ADA criteria or HbA1c levels, IFG, IGT, and IFG + IGT vs. NGT by dysglycemic state, as well as Q4 vs. Q1 by HOMA-IR) at baseline. As a result, the levels of 44 and 39 metabolites significantly differed (*P* < 4.07 × 10^–4^) in PD compared to those in NGT based on the ADA criteria and HbA1c levels, respectively (odds ratios and *P* values are shown in Supplementary Table S2A,B). The levels of 34, 29, and 28 metabolites significantly differed in the IFG, IGT, and IFG + IGT groups, respectively, compared to in NGT by the dysglycemic state (Supplementary Table S2C–E). Finally, the concentrations of 25 metabolites significantly differed in the top quartile (Q4) as the IR group compared to in the lowest quartile (Q1) as referenced by HOMA-IR levels (Supplementary Table S2F).

Elevated glucose and IR are risk indicators for PD development. Thus, we examined metabolite changes in response to increasing fasting glucose, 2 h-PPG, HbA1c, and HOMA-IR levels by multivariable linear regression analysis. Testing of the explanatory fasting glucose and 2 h-PPG revealed 47 and 52 significant metabolites, respectively, associated with dysglycemia (*P* < 4.07 × 10^–4^) (Supplementary Figure S1, Supplementary Table S3A,B). Among them, 33 and 32 metabolites showed consistent results in response to PD using the ADA criteria (75% and 73% of 44 metabolites in Supplementary Table S2A), 32 and 26 metabolites in response to IFG and IGT (94% and 90% of 34 and 29 metabolites in Supplementary Table S2C,D), and 28 and 23 metabolites in response to combined IFG + IGT by logistic regression (100% and 82% of 28 metabolites in Supplementary Table S2E). Furthermore, because increased HbA1c levels and IR are major determinants of the PD risk, linear regression was performed to estimate the difference in PD metabolite changes based on HbA1c and HOMA-IR variables. Consequently, 47 and 37 metabolites were significantly associated with HbA1c and IR after correction for multiple testing, respectively (Supplementary Figure S1, Supplementary Table S3C,D). Among these metabolites, 38 and 22 compositions showed consistent results in response to PD according to HbA1c and the HOMA-IR levels, respectively, and in logistic regression (97% and 88% of 39 and 25 metabolites in Supplementary Table S2B,F). These results indicate that the identified metabolites are reliable for assessing PD and related conditions.

A total of 39 metabolites, which appeared in more than half of previously mentioned results in both logistic and linear regression analysis, were selected as PD-enriched metabolites (Supplementary Table S4). The metabolite dataset contained an acylcarnitine, 10 amino acids, a biogenic amine, 19 glycerophospholipids, 7 sphingolipids, and a hexose. Among these metabolites, 25 were inversely related to the prevalence of PD and included known diabetic markers such as glycine (Gly), kynurenine, lysoPC a C18:2, phosphatidylcholine acyl-alkyl C34:3 (PC ae C34:3) and C44:4 (PC ae C44:4), and sphingomyelin C16:1 (SM C16:1), whereas 14 were positively associated with PD prevalence, including known diabetic markers such as octadecenoylcarnitine (C18:1), alanine (Ala), glutamate (Glu), isoleucine (Ile), phenylalanine (Phe), tyrosine (Tyr), valine (Val), phosphatidylcholine diacyl C36:1 (PC aa C36:1) and C40:5 (PC aa C40:5), and hexose^12,19–22^. Thus, these metabolites were closely linked to the risk of PD.

### Determination of discriminatory metabolites of PD

To improve the accuracy and select more significant metabolites for distinguishing individuals with prediabetic conditions, we performed supervised random forest analysis with the 39 PD-enriched metabolites and 3 covariates. The 30 top-ranked variables, including both metabolites and diabetic risk indicators, were chosen based on the two indices: Mean Decrease Accuracy and Mean Decrease Gini (Supplementary Figure S2). Consequently, 26 metabolites with the potential to discriminate between individuals with prevalent PD and NGT were obtained. Total hexose, which is mainly represented as glucose, was not considered as a variable in subsequent analyses.

### Construction of PD prediction model

We further conducted stepwise regression analysis to select the best prediction model among the discriminatory metabolites. Indeed, 12 independent metabolites, as named the KARE model, maximized the prediction performance at baseline. These metabolites and their associations with prevalent and incident PD, as well as fasting glucose, 2 h-PPG, HbA1c, and HOMA-IR at baseline are listed in Table 2. Six of these (i.e., C18:1, Ala, Met, Val, PC aa C36:1, and SM(OH) C22:2) were linked to an increased risk of prevalent PD, whereas the others (i.e., Gly, Tyr, lysoPC a C18:2, PC ae C30:0, PC ae C42:1, and SM C18:1) were associated with a decreased risk at baseline. Ala, Gly, Val, lysoPC a C18:2, and PC ae C42:1 showed significant altered serum levels in fasting glucose, 2 h-PPG, HbA1c and HOMA-IR from baseline as well as incident PD. However, SM C18:1 showed significance in only fasting glucose, but PC ae C30:0 and SM(OH) C22:2 showed significance in fasting glucose, 2 h-PPG, HbA1c and incident PD. In contrast, Met or Tyr maintained significance in HOMA-IR together with HbA1c or 2 h-PPG, respectively. Finally, PC aa C36:1 was significantly associated with all variables but not with incident PD. Next, we examined whether the predictive ability of these metabolites was comparable to that of previously established clinical parameters at baseline by analyzing the AUC (Supplementary Figure S3). The KARE model used to discriminate between prevalent PD and NGT had an AUC of 0.84 (95% confidence interval: 0.82, 0.86), which was higher than that of the FHS and KORA metabolites and traditional CRF models [AUC 0.74 (0.71, 0.76), 0.66 (0.63, 0.69), and 0.74 (0.72, 0.76), respectively, *P* < 0.0001] and lower than that of the fasting glucose (Glu0) model [AUC 0.95 (0.94, 0.96), *P* < 0.0001], which was highly correlated with the diabetic condition (Supplementary Table S5). Notably, Val and Tyr as well as Gly and lysoPC a C18:2, previously reported by FHS and KORA, were selected in the KARE model. When the KARE model was combined with the established risk prediction models, i.e., CRF and Glu0, the discrimination was slightly but significantly improved according to the AUC of 0.88 (0.86, 0.89) and 0.96 (0.95, 0.97), respectively, (*P* < 0.001).

**Table 2:** Association of stepwise regression selected 12 metabolites with prevalent and incident PD, as well as fasting glucose, 2 h-PPG, HbA1c, and HOMA-IR at baseline (*P* < 0.05). Odds ratio (OR) and 95% confidence interval (CI) estimates provided from logistic regression and Beta (beta coefficient; effect size) from linear regression in the normalized metabolites.

	Metabolites	Prevalent PD		Incident PD		Fasting glucose		2 h-PPG		HbAlc	HOMA-IR		
		OR (95% CI)	*P* value	OR (95% CI)	*P* value	Beta (95% CI)	*P* value	Beta (95% CI)	*P* value	Beta (95% CI)	*P* value	Beta (95% CI)	*P* value
**Acylcarnitines (1)**													
1	C18:1	1.67 (1.47, 1.91)	2.36E−14	1.05 (0.84, 1.32)	0.66	0.18 (0.14, 0.22)	2.45E−16	0.10 (0.06, 0.14)	9.75E−06	0.00 (-0.05, 0.04)	0.97	0.01 (-0.04, 0.05)	0.77
**Amino acids (5)**													
2	Ala	1.73 (1.47, 2.02)	1.29E−11	1.42 (1.08, 1.88)	1.22E−02	0.21 (0.16,0.27)	1.53E−15	0.19 (0.14, 0.24)	1.09E−11	0.12 (0.07, 0.18)	1.02E−05	0.19 (0.13, 0.24)	3.22E−11
3	Gly	0.50 (0.43, 0.58)	3.01E−20	0.71 (0.56, 0.90)	6.00E−03	-0.23 (-0.28, -0.19)	2.00E−16	-0.22 (-0.27, -0.18)	2.00E−16	-0.12 (-0.17, -0.07)	9.69E−07	-0.17 (-0.22, -0.13)	4.39E−12
4	Met	1.23 (1.05, 1.44)	1.20E−02	0.95 (0.68, 1.32)	0.75	0.01 (-0.05, 0.05)	0.97	0.05 (-0.01, 0.10)	0.10	0.07 (0.01, 0.12)	1.89E−02	-0.11 (-0.16, -0.05)	2.27E−04
5	Tyr	0.84 (0.70, 0.99)	4.43E−02	1.10 (0.78, 1.54)	0.58	0.02 (-0.04, 0.08)	0.51	-0.10 (-0.16, -0.04)	9.33E−04	-0.04 (-0.10, 0.02)	0.23	0.17 (0.11, 0.24)	8.50E−08
6	Val	1.91 (1.61, 2.26)	5.28E−14	1.47 (1.11, 1.94)	6.77E−03	0.17 (0.11, 0.22)	3.86E−09	0.23 (0.17, 0.28)	9.40E−15	0.16 (0.10, 0.21)	1,71E−07	0.11 (0.05, 0.16)	3.51E−04
**Glycerophospholipids (4)**													
7	lysoPC a C18:2	0.61 (0.53, 0.69)	1.03E−13	0.59 (0.46, 0.74)	1.50E−05	-0.07 (-0.11, -0.02)	2.11E−03	-0.25 (-0.29, -0.20)	2.00E−16	-0.20 (-0.24, -0.15)	2.00E−16	-0.11 (-0.16, -0.06)	2.68E−06
8	PC aa C36:1	1.79 (1.53, 2.11)	7.38E−13	1.07 (0.79, 1.54)	0.68	0.11 (0.06, 0.16)	3.49E−05	0.09 (0.04, 0.15)	6.08E−04	0.24 (0.18, 0.29)	2.00E−16	0.10 (0.05, 0.16)	2.23E−04
9	PC ae C30:0	0.59 (0.51, 0.68)	1.05E−12	0.62 (0.48, 0.81)	4.02E−04	-0.09 (-0.14, -0.05)	1.06E−04	-0.10 (-0.15, -0.06)	2.15E−05	-0.17 (-0.22, -0.12)	1.09E−11	-0.02 (-0.07, 0.03)	0.45
10	PC ae C42:1	0.56 (0.48, 0.66)	6.60E−12	0.76 (0.58, 1.00)	5.00E−02	-0.14 (-0.19, -0.09)	1.01E−07	-0.12 (-0.18, -0.07)	3,55E−06	-0.15 (-0.21, -0.10)	2.20E−08	-0.15 (-0.21, -0.10)	2.65E−08
**Sphingolipids (2)**													
11	SM C18:1	0.65 (0.53, 0.80)	5.58E−05	0.85 (0.60, 1.21)	0.37	-0.18 (-0.24, -0.11)	7.67E−08	0.04 (-0.02, 0.11)	0.21	-0.04 (-0.11, 0.03)	0.30	-0.01 (-0.08, 0.06)	0.81
12	SM (OH) C22:2	1.65 (1.31, 2.08)	1.74E−05	1.92 (1.31, 2.84)	9.33E−04	0.09 (0.01, 0.16)	1.93E−02	0.11 (0.04, 0.19)	3.79E−03	0.14 (0.07, 0.22)	3.11E−04	0.05 (-0.02, 0.13)	0.17

### Predicting incident PD with the KARE model

**Figure 1:**
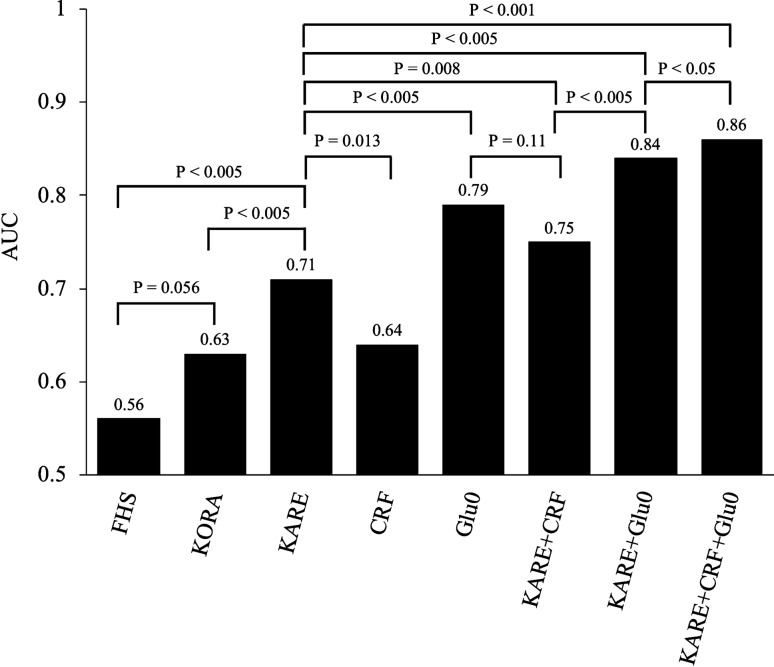
Comparison of AUCs from different prediction models for PD incidence in the follow-up dataset determined by DeLong’s test. *FHS* Framingham Heart Study model (Leucine, Isoleucine, Valine, Tyrosine, and Phenylalanine), *KORA* KORA cohort model (Glycine, lysoPC a C18:2 and acetylcarnitine), *CRF* Clinical Risk Factors (Age, Sex, BMI, HDL-C, LDL-C and TG), *Glu0* fasting glucose.

Figure 1 shows the AUC comparisons across the different prediction models in the follow-up dataset. KARE metabolites discriminated future PD with an AUC of 0.71 (95% confidence interval: 0.67, 0.76), which was significantly higher than that of the FHS and KORA metabolite models [AUC 0.56 (0.50, 0.60) and 0.63 (0.58, 0.68), *P* < 0.005, respectively]. The AUC of the traditional CRF model was significantly lower than that of the KARE model [AUC 0.64 (0.59, 0.69), *P* < 0.013]. However, as expected, the AUC of Glu0 model was significantly higher than that of the KARE model [AUC 0.79 (0.76, 0.84), *P* < 0.005]. The AUC of the combined KARE and CRF models significantly improved the predictive performance compared to KARE or CRF model only [0.75 (0.71, 0.80), *P* < 0.008]. More importantly, adding Glu0 to the combined CRF and KARE model maximized the AUC to 0.86 (0.82, 0.89). These results indicate that the selected metabolite model improved the prediction of PD compared to other prediction models and established clinical parameters.

**Figure 2:**
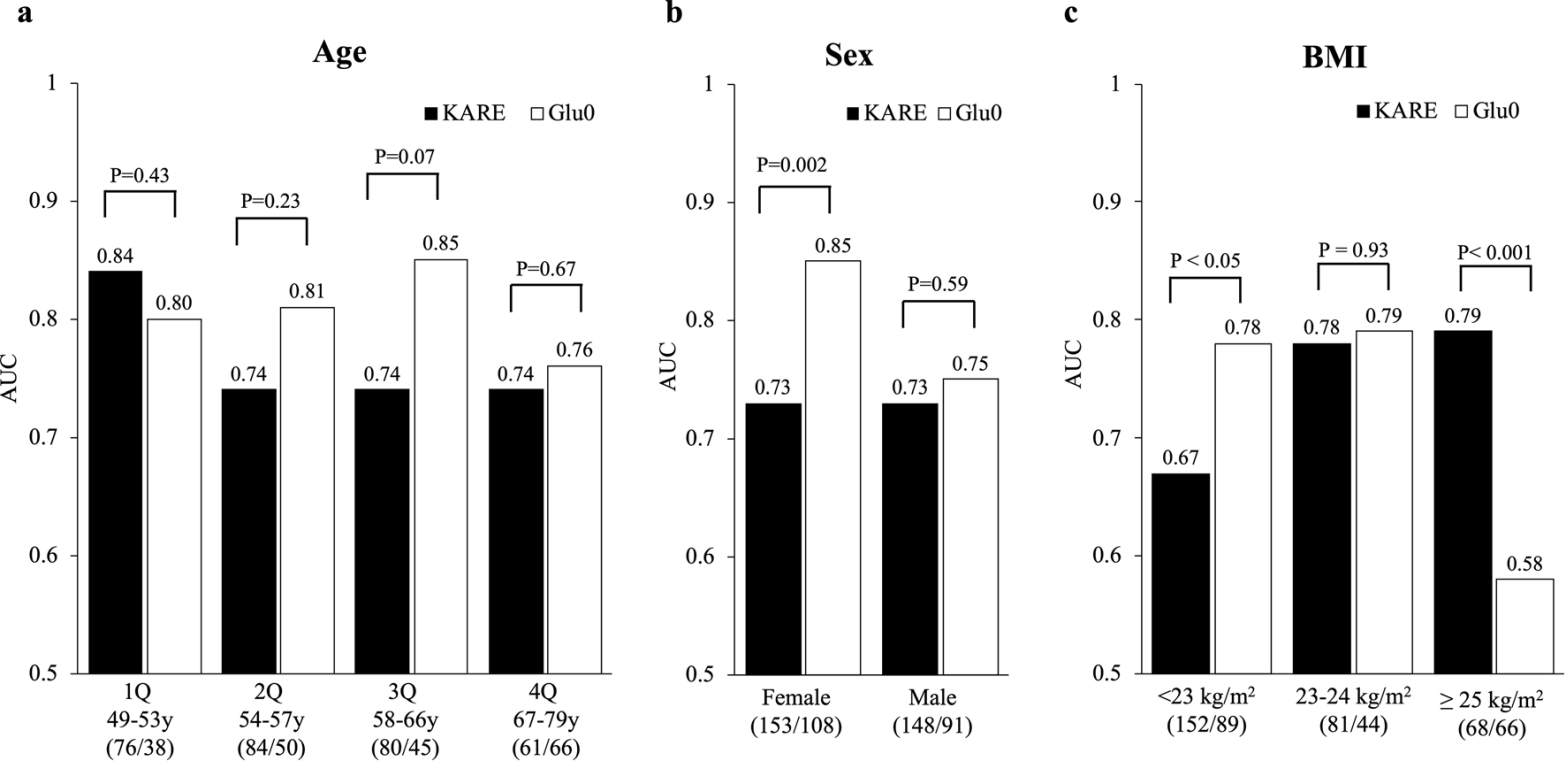
Comparisons of AUCs in the follow-up dataset according to age (**a**), sex (**b**), and BMI (**c**) groups. DeLong’s test was performed to compare AUCs between the KARE and fasting glucose (Glu0) prediction models. (/): number of controls and incident cases analyzed in the follow-up.

We also tested whether the metabolite and fasting glucose models predicted the risk of incident PD in different baseline risk groups. The AUC values of the KARE metabolite and fasting glucose models in subpopulations stratified by age, sex, and BMI (used for covariates) are shown in Fig. 2. In the four age quartile groups, the AUC of the KARE model did not significantly differ from that of Glu0 model but was higher in early ages and smaller in advancing ages. The AUC of the Glu0 model was significantly higher than that of the KARE model in the female group [AUC 0.85 (0.80, 0.90) vs. 0.73 (0.66, 0.79), *P* < 0.005], but showed no significant difference in the male group. Similarly, in the group with a normal BMI (< 23 kg/m^2^), the AUC of the Glu0 model was significantly higher than that of the KARE model [AUC 0.78 (0.71, 0.84) vs. 0.67 (0.60, 0.74), *P* < 0.05]. In contrast, in the overweight BMI (23 to 24 kg/m^2^) no significant difference was observed [AUC 0.78 (0.70, 0.87) vs. 0.79 (0.71, 0.87), *P* = 0.93], whereas in the obese BMI group (≥ 25 kg/m^2^), the AUC of the KARE model was significantly higher than that of the Glu0 model [AUC 0.79 (0.72, 0.87) vs. 0.58 (0.48, 0.68), *P* < 0.001]. These results suggest that the KARE metabolite model is useful for predicting PD in obese individuals.

## Discussion

PD is considered as an important risk factor for the development of T2D as well as vascular problems, kidney disease, and nerve and retinal injuries. Thus, an early PD diagnosis can improve the quality of life of patients suffering from T2D and related complications. However, because approximately 90% of patients with PD are not aware of their conditions until they are diagnosed with diabetes, the development of suitable prediction models using effective PD-related biomarkers in biofluids, including blood, serum, plasma, and urine is urgently required.

Using targeted metabolomics combined with statistical analysis enable identification of metabolic markers for discriminating between the absence and presence of PD in a cross-sectional study and perform predictive analysis of incident PD in a 6-year follow-up study. As a result, 39 metabolites were significantly associated with the risk of PD in different biochemical phenotypic traits. Finally, from the results of both random forest and stepwise logistic regression analyses, 12 metabolites were further selected as a KARE model, including an acylcarnitine, five amino acids, four glycerophospholipids, and two sphingolipids, because of their powerful discrimination for predicting the future onset of PD. The KARE metabolite model significantly improved the PD prediction performance compared to the FHS or KORA metabolite model, CRF, or fasting glucose. We also showed that the combined metabolite model predicted future PD better than fasting glucose in the obese population. In addition, we concluded from the FHS and KORA studies that Val, Tyr, Gly, and lysoPC a C18:2 are predictors of diabetes development independently of other risk factors^12,15^. Among the other KARE metabolites, C18:1, Ala, Met, PC aa C36:1, and SM(OH) C22:2 were previously reported as potential predictive and diagnostic markers for PD or T2D. As a free fatty acid, the plasma levels of octadecenoylcarnitine (C18:1) were increased not only in T2D but also in IGT and IFG, and may lead to hyperglycemia by excessive IR-induced beta oxidation^22,23^. Alanine (Ala) is a gluconeogenic substrate linked to impaired insulin sensitivity prior to the elevation of fasting glucose or insulin levels and may predict the incidence of diabetes as a marker of attenuated glucose tolerance^24,25^. An increase in circulating blood methionine (Met) and altered Met catabolism led to excessive conversion of Met into *S*-adenosylmethionine by a liver-specific Met adenosyltransferase, and thus to diabetes pathogenesis (insulin-resistant and β-cell dysfunctions)^9,26,27^. By controlling Met levels, the conditions of glucose homeostasis, insulin sensitivity, and oxidative stress with activation of the fibroblast growth factor 21 and protein phosphatase 2A signals in diabetes may improve^28,29^. As previously reported, increased or reduced concentrations of PC aa C36:1 or SM(OH) C22:2 in plasma indicated the incidence of T2D^12,20^. Significant concentration changes in these metabolites were correspondingly observed in our study, suggesting that KARE metabolite levels may change in T2D as well as PD, supporting the relevance that the metabolites were linked to diabetes progression. As shown in Table 2, all metabolites at baseline were associated with prevalent PD, although some were not associated with incident PD in the follow-up. However, we retained all metabolites in the KARE prediction model, as this lack of association may have resulted from the small size effect, and only a large sample may generate sufficient statistical power to obtain significant results. Another reason may be that sequential metabolite changes take place depending on the PD duration and progression, which could not be controlled in statistical analysis. Addition of KARE metabolites complemented the PD prediction based on fasting glucose and CRFs, and thus when combined, yielded the best model, as shown in Fig. 1. This may be attributed to the selection of the KARE model which could be performed independently of CRFs, possibly because the significant metabolites were linked to other causes of PD. Furthermore, the improvement in the predictive performance with additional metabolites implies that metabolic alterations in PD besides impaired glucose regulation were involved. According to Fig. 2, the AUC of the metabolite model was higher than that of the fasting glucose model in the obese subgroup but was not significantly changed in the male and ages subgroups. This result suggests that the metabolite model had better or equal accuracy in PD prediction in those with no obvious traditional risk factors. However, when the AUC of the metabolite model was lower than that of the fasting glucose model in the female and leaner subgroups, further analysis was required to identify additional metabolites with good prediction performance.

In this study, we detected a difference in the metabolic profiles of PD and NGT groups and evaluated PD prediction in cross-sectional and follow-up studies. However, because of the broad metabolite spectrum in this study, validation of the selected metabolite model has not been performed in different cohorts. Another limitation is that there were no clear clues for the metabolic alterations of the prediction model that could occur before PD onset. Nevertheless, a series of metabolic alterations may occur before the onset of PD, based on the beta values of the linear regression with fasting and postprandial glucose, HbA1c, or HOMA-IR. Furthermore, compared to our previous report, most of these metabolites, C18:1, Ala, Val, Gly, lysoPC a C18:2, PC aa C36:1, PC ae C42:1, and SM C18:1, were also significantly altered in subjects with T2D compared to in non-patients^30^, indicating that the metabolites are involved in the overall diabetes progression.

In conclusion, introduction of a metabolomics approach to identify serum metabolites yielded a novel prediction model containing 12 metabolites related to PD, and thus improved the prediction performance of PD in combination with diabetes risk factors. These findings may improve the understanding of the PD metabolic etiology and promote the prevention of this and other related diseases.

## Methods

### Study subjects and sampling

The KARE cohort for the prospective population study was established by the Korean Genome and Epidemiology Study (KoGES) in the Ansan and Ansung areas of South Korea^31^. The KARE cohort was assembled and biannually surveyed from 2001 to 2014 through a twelve-year follow-up. KARE 2nd survey (KARE S2) was administered to 7515 individuals examined from 2005 to 2006. In total, 1723 subjects, including 799 PD and 924 NGT individuals, were recruited from the KARE S2 cohort for cross-sectional study as a baseline (Table 1). The follow-up data collection of the KARE 5th survey (KARE S5) took place from 2011 to 2012 (6 years after baseline data collection). Of the 924 NGT individuals at KARE S2, 500 were followed up for 6 years, resulting in 199 incidences of PD and 301 remaining as NGT; the prediction analysis was performed on this population (Table 1). According to the criteria of the American Diabetes Association (ADA)^1^, NGT was defined as fasting glucose levels below 100 mg/dL, 2-h postprandial glucose (2 h-PPG) levels below 140 mg/dL, and glycosylated hemoglobin A1c (HbA1c) levels below 5.7%. However, fasting glucose levels of 100–125 mg/dL, 2 h-PPG levels of 140–199 mg/dL, or HbA1c levels of 5.7 to 6.4% were defined as PD.

### Metabolite measurements and quality assessment

Serum metabolites in subjects from both the KARE S2 (n = 1723) and KARE S5 (n = 500) were quantitatively analyzed by a targeted metabolomics approach using the AbsoluteIDQ p180 kit (BIOCRATES Life Science, Innsbruck, Austria), as previously described in our studies^30^. Briefly, to quantify metabolites in the serum samples, liquid chromatography system and flow-injection analysis-mass spectrometry were performed using an API 4000 QTRAP system (Applied Biosystems, Foster City, CA, USA), equipped with an Agilent 1200 HPLC system (Agilent Technologies, Santa Clara, CA, USA) and following the manufacturer’s instructions. The concentrations of 186 metabolites, 40 acylcarnitines, 21 amino acids, 19 biogenic amines, 90 glycerophospholipids, 15 sphingolipids, and a hexose were measured in micromolar units with the MetVal software package (BIOCRATES Life Sciences). Quality control (QC) was performed by using calibration standards and QC samples included in the kit, and reference standards were used as normal human pooled serum. Data quality of each metabolite was checked based on the following criteria. First, the coefficient of variance for each metabolite in the reference standards < 15%; second, half of the analyzed metabolites concentrations in the reference standards > limit of detection (LOD), which was set to three times the median of the three blank samples within each kit plate; and third, half of the analyzed metabolite concentrations in the experimental samples > LOD. After excluding metabolites that did not meet the quality criteria, subsequent analyses were performed using 123 and 131 metabolites in the KARE S2 baseline and KARE S5 follow-up, respectively.

### Statistical analyses

**Figure 3:**
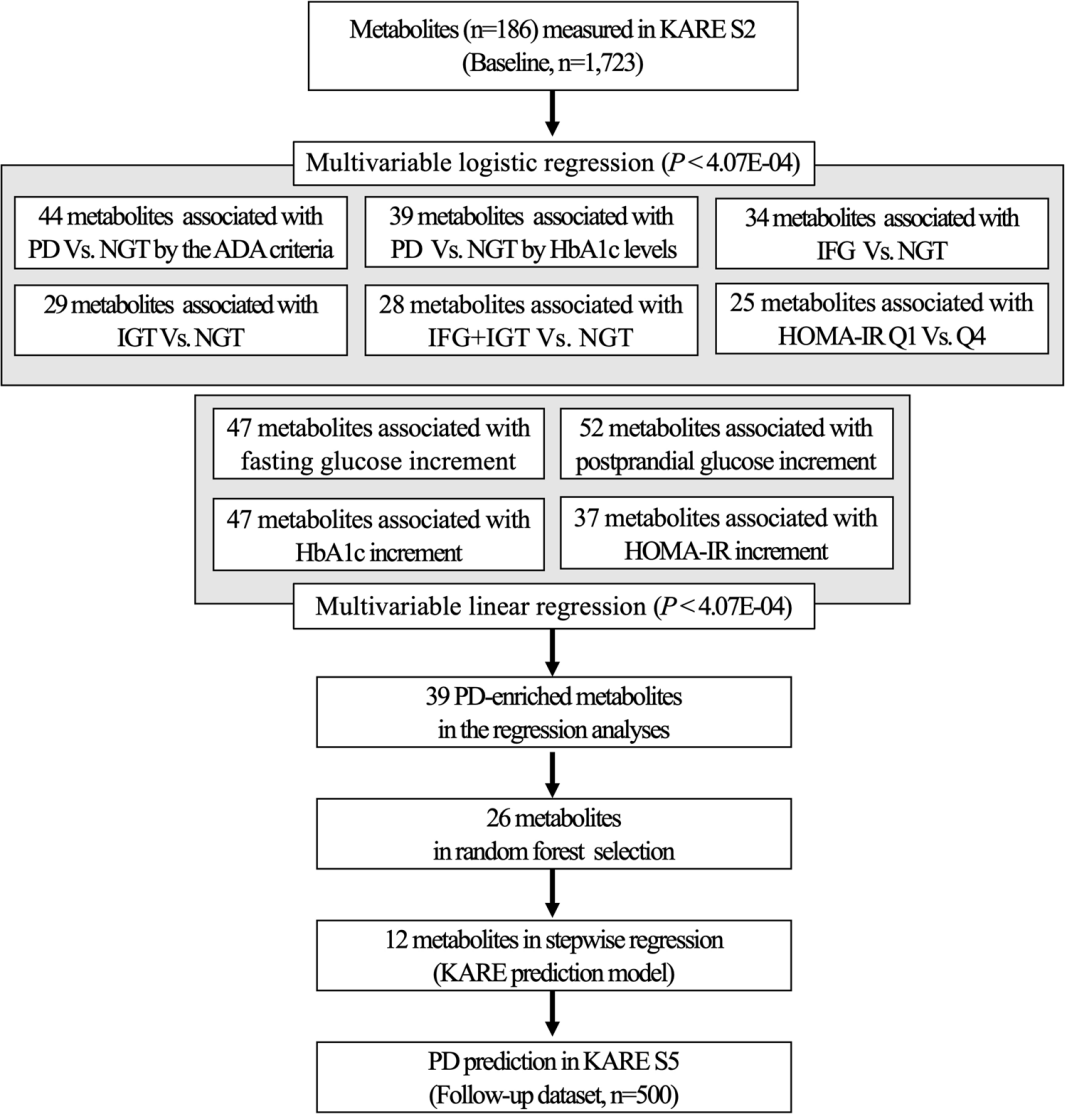
Flow chart of metabolite selection. The selected metabolites showed significant concentration differences in multivariable logistic and linear regression analysis with adjustment for age, sex, and BMI. The Bonferroni correction was applied for multiple testing with *P* < 4.07E−04. *KARE S2/S5* Korea Association REsource (KARE) cohort 2nd Survey/5th Survey, *PD* Prediabetes, *ADA* American Diabetes Association, *HbA1c* Glycated Hemoglobin, *IFG* impaired fasting glucose, *IGT* impaired glucose tolerance, *HOMA-IR Q1/Q4* homeostasis model assessment of insulin resistance 1st quartile/4th quartile.

Statistical analyses were conducted with R statistical package environment (http://www.r-project.org) and SPSS for windows (version 20.0, IBM). The concentration of each metabolite was log-transformed and then the z-score was normalized to a mean equal to zero and variance of one. Figure 3 shows the selection of metabolites. First, we tested the association between the 123 individual metabolites and different prediabetic phenotypes by multivariable regression analysis adjusting for age, sex, and BMI in the KARE S2 baseline. To perform the regression analysis, 1723 subjects with PD, defined by ADA criteria, were stratified in subgroups according to their PD-related biochemical traits, such as IFG, IGT, combined IFG/IGT, HbA1c, and homeostasis model assessment of insulin resistance (HOMA-IR) as follows: (1) NGT and PD groups by ADA criteria (Table 1); (2) NGT, IFG, IGT, and combined IFG/IGT groups based on glucose levels (Supplementary Table S1A); (3) NGT and PD groups based on HbA1c levels (Supplementary Table S1B); and (4) quartile groups based on HOMA-IR levels (Supplementary Table S1C). HOMA-IR was calculated as follows: fasting insulin (μU/mL) × ***fasting glucose (mg/dL)/405^32^. Multivariable regression analyses were performed using the glm() and lm() function for the logistic and linear model with the MASS R package. To control the false discovery rates from multiple testing, we applied stringent Bonferroni corrections at a level of significance of 4.07 × 10^–4^ (5% level and 123 metabolites: 0.05/123). As a result, 39 significant metabolites were selected as PD-enriched metabolites that appeared more than five times in the 10 regression analyses. Second, we employed the random forest algorithm to select differentially abundant metabolites between the NGT and PD groups by using the ‘randomForest’ package^33^. The classification method based on the variable importance scores identified the 26 highest ranked metabolites from the PD-enriched metabolites by excluding less important metabolite classifiers. Third, a stepwise backward elimination logistic regression was applied to select metabolites with maximum prediction performance. As a result, the 12 most significant predictable metabolites were selected and included in a final model with minimal Akaike’s information criterion.

### Prediction of PD incidence in the follow-up dataset

The PD incidence was predicted in the KARE S5 follow-up dataset using the predictive model including 12 metabolites and previously reported models from other studies. Analysis of the area under the receiver operator characteristic (ROC) curve (AUC), based on predicted probabilities from a logistic regression without stepwise variable selection, was performed to compare different prediction models. The following models were compared: KARE metabolite model (metabolites selected in the current study), Framingham Heart Study (FHS) metabolite model (amino acids reported by the FHS research; BCAAs, tyrosine, and phenylalanine)^15^, KORA metabolite model (glycine, lysoPC a C18:2, acetylcarnitine)^12^, and traditional CRFs (age, sex, BMI, HDL-C, LDL-C, and TG) and fasting glucose single and combined models. We also tested the models according to age (quartile groups), sex (male/female), and BMI classification for Asians [normal (< 23 kg/m^2^), overweight (23–24.9 kg/m^2^), and obese (≥ 25 kg/m^2^)], as previous studies showed a broad influence of age, sex, and BMI on the serum metabolome in the general and diabetic populations^25,34^. A *P* < 0.05 obtained from the DeLong's test of the difference between paired AUC was used as a cut off to detect significant improvement across the models. Comparison of the AUC values from ROC analyses were conducted using the roc.test() of the pROC package in R.

### Ethical approval and informed consent

This study was approved by the Korea National Institute of Health Institutional Review Board (Approval Number: 2017-03-01-P-A) and was performed in accordance with relevant guidelines and regulations. Written informed consent was obtained from all enrolled participants.

## Supplementary Information

Supplementary Information

## Data Availability

The datasets generated and/or analyzed in the current study have been included in this article or supplementary files. Additional raw and processed data have been uploaded to the Open Science Framework (https://osf.io/zfsrd).
