# Supplementary material for: Identification of metabolic markers predictive of prediabetes in a Korean population
Source: Sci Rep. 2020 Dec 15;10:22009. doi: 10.1038/s41598-020-78961-4 (PMC7738529; doi:10.1038/s41598-020-78961-4)
Supplement: Supplementary file 1 — Supplementary Information [file 41598_2020_78961_MOESM1_ESM.docx]

**Identification of metabolic markers predictive of prediabetes in a Korean population**

Heun-Sik Lee, Tae-Joon Park, Jeong-Min Kim, Jun Ho Yun, Ho-Yeong Yu, Yeon-Jung Kim, Bong-Jo Kim*****

Division of Genome Research, Center for Genome Science, National Institute of Health, Osong Health Technology Administration Complex, 187, Osongsaengmyeong 2-ro, Osong-eup, Heungdeok-gu, Cheongju-si, Chungchenogbuk-do, 28159, Republic of Korea

**Supplementary Figure S1**. Venn diagram of metabolites. The Venn diagram is a representation of sample-dependent numbers of shared and unique metabolites in response to increments of fasting glucose, 2h-PPG, HbAlc, and HOMA-IR levels based on multivariable linear regression analysis. The metabolites are listed in Supplementary Table S3.


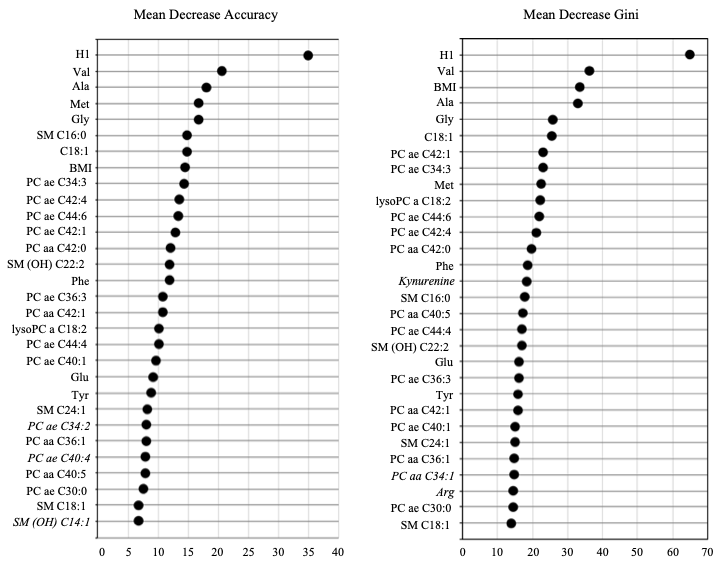


**Supplementary Figure S2**. Random forest selection with PD-enriched metabolites and covariates (i.e., Age, Sex, and BMI). 26 highest ranking variables (regular) are denoted by the mean decrease in classification accuracy and the mean decrease of Gini importance score. Italic: different variables between the two indices.

**
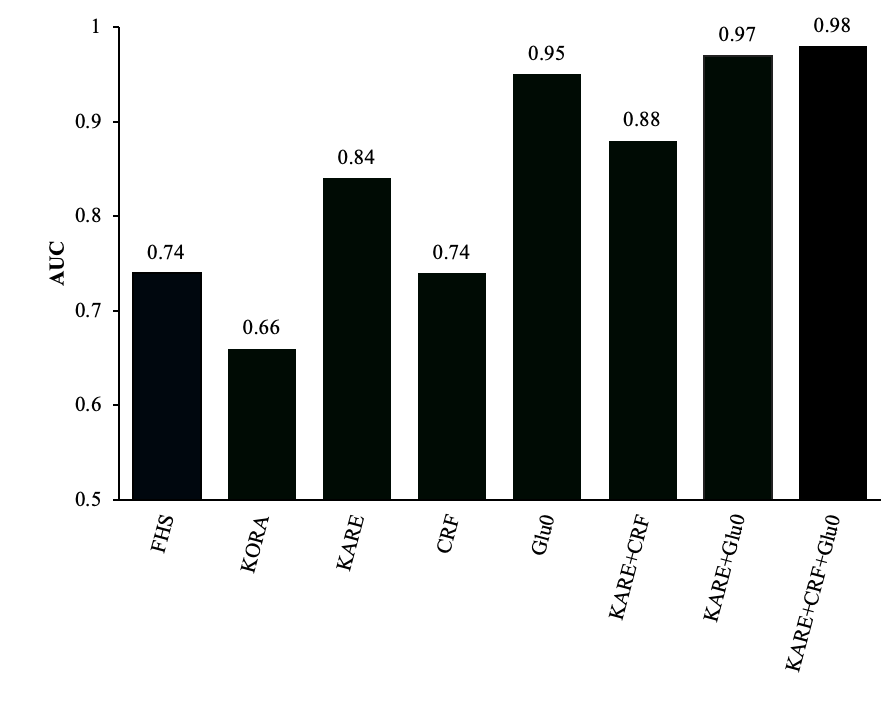
**

**Supplementary Figure S3**. Prediction of prevalent PD with KARE model containing 12 metabolites at the baseline dataset. The AUC for prediction of PD was calculated as the area under the curve of different prediction models. FHS; Framingham Heart Study model containing leucine, isoleucine, valine, tyrosine and phenylalanine), KORA; KORA cohort model containing glycine, lysophosphatidylcholine 18:2 and acetylcarnitine, CRF; Clinical Risk Factors containing age, sex, BMI, HDL, LDL and TG, Glu0; fasting glucose.

**Supplementary Table S1.** Clinical and laboratory parameters in different prediabetic subtypes from the baseline population. Each subtype is defined by dysglycemic state, HbA1c, and insulin resistance (HOMA-IR) levels. (a) Characteristic of the study population, NGT, IFG, IGT, and combined IFG+IGT, grouped by according to dysglycemic state. (b) Characteristics of the study population, NGT and PD, grouped by according to HbA1c levels. (c) Characteristics of the study population, four quartiles, grouped by according to HOMA-IR levels.

**a**

| Clinical & laboratory parameters | Baseline (n=1,723) | | | |
| --- | --- | --- | --- | --- |
|  | NGT (n=997) | IFG (n=255) | IGT (n=265) | IFG+IGT (n=206) |
| Age (years) | 54.93±8.74 | 56.09±8.80 | 58.38±8.54 | 54.80±8.31 |
| Sex (female) (%) | 55.07 | 30.99 | 63.02 | 38.84 |
| BMI (kg/m^2^) | 23.42±2.97 | 25.15±3.10 | 25.46±3.31 | 25.62±2.92 |
| HDL cholesterol (mg/dl) | 45.26±10.16 | 43.34±10.84 | 43.19±9.32 | 43.90±10.88 |
| LDL cholesterol (mg/dl) | 119.31±30.21 | 120.99±31.85 | 128.45±33.24 | 121.78±31.34 |
| Triglycerides (TG) (mg/dl) | 112.64±58.31 | 136.76±65.37 | 154.29±71.94 | 147.50±70.84 |
| HbA1c (%) | 5.31±0.33 | 5.57±0.35 | 5.88±0.18 | 5.71±0.37 |
| Fasting glucose (mg/dl) | 83.69±5.37 | 105.22±5.11 | 90.92±5.73 | 106.16±4.99 |
| 2h-PPG (mg/dl) | 91.65±18.79 | 108.26±21.90 | 163.35±16.26 | 166.73±17.30 |
| Fasting insulin (μU/ml) | 6.77±3.11 | 8.67±4.34 | 8.36±3.80 | 9.06±5.16 |
| HOMA-IR | 1.41±0.68 | 2.25±1.15 | 1.88±0.88 | 2.38±1.41 |

Data presented as mean ± deviations (SD) and n (%). BMI; body mass index, HDL; high density lipoprotein, LDL; low-density lipoprotein, HbA1c; glycated hemoglobin. 2 h-PPG; 2 h-postprandial glucose. HOMA-IR; homeostasis model assessment of insulin resistance (Fasting insulin (μU/ml) x Fasting glucose (mg/dL)/405), IFG; Fasting glucose level of 100 to 125 mg/dL, IGT; 2h-PPG level of 140 to 199 mg/dL, IFG+IGT; combined IFG and IGT.

**b**

| Clinical & laboratory parameters | Baseline (n=1,723) | |
| --- | --- | --- |
|  | NGT (n=1,105, HbA1c < 5.7) | PD (n=618, 5.7≤HbA1c ≤6.4) |
| Age (years) | 54.24±8.56 | 58.07±8.55 |
| Sex (female) (%) | 48.14 | 55.50 |
| BMI (kg/m^2^) | 23.71±2.99 | 25.21±3.31 |
| HDL cholesterol (mg/dl) | 45.51±10.72 | 42.68±9.10 |
| LDL cholesterol (mg/dl) | 118.39±30.45 | 126.35±31.88 |
| Triglycerides (TG) (mg/dl) | 115.66±59.46 | 146.17±70.82 |
| HbA1c (%) | 5.24±0.24 | 5.90±0.19 |
| Fasting glucose (mg/dl) | 87.96±10.36 | 95.52±9.86 |
| 2h-PPG (mg/dl) | 98.68±27.35 | 141.71±35.33 |
| Fasting insulin (μU/ml) | 7.15±3.72 | 8.32±3.91 |
| HOMA-IR | 1.58±0.95 | 1.98±0.99 |

**c**

| Clinical & laboratory parameters | Baseline (n=1,723) | | | |
| --- | --- | --- | --- | --- |
|  | Q1 (n=431) | Q2 (n=431) | Q3 (n=430) | Q4 (n=431) |
| Age (years) | 55.61±9.02 | 55.66±8.92 | 55.18±8.28 | 56.01±8.78 |
| Sex (female) (%) | 44.32 | 51.28 | 53.95 | 53.60 |
| BMI (kg/m^2^) | 22.45±2.74 | 23.52±2.78 | 24.68±2.85 | 26.35±3.01 |
| HDL cholesterol (mg/dl) | 46.73±10.44 | 45.07±10.68 | 44.50±10.23 | 41.68±8.99 |
| LDL cholesterol (mg/dl) | 116.98±30.96 | 120.10±31.02 | 123.94±30.70 | 124.08±31.67 |
| Triglycerides (TG) (mg/dl) | 106.11±55.03 | 118.44±61.20 | 126.38±62.14 | 156.61±71.98 |
| HbA1c (%) | 5.36±0.37 | 5.39±0.37 | 5.54±0.38 | 5.64±0.36 |
| Fasting glucose (mg/dl) | 83.62±8.22 | 87.72±8.77 | 92.79±9.79 | 98.57±10.12 |
| 2h-PPG (mg/dl) | 98.03±31.40 | 106.28±33.16 | 120.73±37.16 | 131.43±35.90 |
| Fasting insulin (μU/ml) | 4.39±0.99 | 6.04±0.66 | 7.62±0.99 | 12.23±4.69 |
| HOMA-IR | 0.90±0.19 | 1.30±0.10 | 1.73±0.17 | 2.97±1.19 |

Q1; first quartile, Q2; second quartile, Q3; third quartile, Q4: fourth quartile

**Supplementary Table S2.** Significantly change in metabolite levels due to PD defined by the ADA criteria, HbA1c, dysglycemic states, and insulin resistance (HOMA-IR) levels. (a) Odds ratio (OR) and *P*-value in a pairwise comparison between PD (n=799) and NGT (n=924) by the ADA criteria in the KARE S2 baseline (total, n=1,723). 44 metabolites show significant concentration differences in the comparison using multivariable logistic regression analysis (after the Bonferroni correction for multiple testing with *P* < 4.07E-04) with adjustment for age, sex, and BMI. (b) OR and *P*-value in a pairwise comparison between PD (n=618) and NGT (n=1,105) by HbA1c levels in the KARE S2 baseline (total, n=1,723). 39 metabolites show significant concentration differences in the comparison. (c) OR and *P*-value in a pairwise comparison between IFG (n=255) and NGT (n=997) in the KARE S2 baseline (total, n=1,252). 34 metabolites show significant concentration differences in the comparison. (d) OR and *P*-value in a pairwise comparison between IGT (n=265) and NGT (n= 997) in the KARE S2 baseline (total, n=1,262). 29 metabolites show significant concentration differences in the comparison. (e) OR and *P*-value in a pairwise comparison between IFG+IGT (n=206) and NGT (n=997) in the KARE S2 baseline (total, n=1,203). 28 metabolites show significant concentration differences in the comparison. (f) OR and *P*-value in a pairwise comparison between the top quartile (Q4, n=431) as IR and lowest quartile (Q1, n=431) as reference by HOMA-IR levels in the KARE S2 baseline (total, n=862). 25 metabolites show significant concentration differences in the comparison.

**a**

|  | Metabolites | | PD Vs. NGT (by the ADA criteria) | |
| --- | --- | --- | --- | --- |
| # | Abbreviation | Biochemical name | OR (95% CI), per SD | *P-*value |
| 1 | C16 | Hexadecanoylcarnitine | 1.24 (1.11-1.38) | 1.04E-04 |
| 2 | C18:1 | Octadecenoylcarnitine | 1.41 (1.26-1.57) | 3.24E-09 |
| 3 | Ala | Alanine | 1.65 (1.47-1.84) | 6.72E-18 |
| 4 | Glu | Glutamate | 1.43 (1.28-1.59) | 3.21E-10 |
| 5 | Gly | Glycine | 0.73 (0.65-0.81) | 9.31E-09 |
| 6 | Lys | Lysine | 1.29 (1.16-1.44) | 2.00E-06 |
| 7 | Met | Methionine | 1.30 (1.16-1.45) | 5.00E-06 |
| 8 | Pro | Proline | 1.34 (1.20-1.50) | 1.84E-07 |
| 9 | Tyr | Tyrosine | 1.27 (1.13-1.41) | 2.40E-05 |
| 10 | Val | Valine | 1.69 (1.50-1.91) | 5.78E-18 |
| 11 | Kynurenine | Kynurenine | 0.79 (0.71-0.88) | 1.10E-05 |
| 12 | Putrescine | Putrescine | 0.79 (0.71-0.87) | 8.00E-06 |
| 13 | Taurine | Taurine | 1.21 (1.10-1.35) | 2.31E-04 |
| 14 | lysoPC a C18:2 | lysoPhosphatidylcholine acyl C18:2 | 0.70 (0.62-0.78) | 4.41E-10 |
| 15 | PC aa C34:1 | Phosphatidylcholine diacyl C34:1 | 1.32 (1.19-1.47) | 3.37E-07 |
| 16 | PC aa C34:4 | Phosphatidylcholine diacyl C34:4 | 1.29 (1.16-1.43) | 3.00E-06 |
| 17 | PC aa C38:0 | Phosphatidylcholine diacyl C38:0 | 0.81 (0.73-0.90) | 9.80E-05 |
| 18 | PC aa C38:1 | Phosphatidylcholine diacyl C38:1 | 0.81 (0.73-0.89) | 4.50E-05 |
| 19 | PC aa C38:5 | Phosphatidylcholine diacyl C38:5 | 1.35 (1.22-1.50) | 1.85E-08 |
| 20 | PC aa C42:0 | Phosphatidylcholine diacyl C42:0 | 0.64 (0.57-0.71) | 2.38E-15 |
| 21 | PC aa C42:1 | Phosphatidylcholine diacyl C42:1 | 0.67 (0.60-0.74) | 3.80E-13 |
| 22 | PC ae C34:2 | Phosphatidylcholine acyl-alkyl C34:2 | 0.75 (0.68-0.84) | 1.59E-07 |
| 23 | PC ae C34:3 | Phosphatidylcholine acyl-alkyl C34:3 | 0.63 (0.57-0.71) | 3.02E-16 |
| 24 | PC ae C36:3 | Phosphatidylcholine acyl-alkyl C36:3 | 0.69 (0.62-0.76) | 5.96E-12 |
| 25 | PC ae C38:4 | Phosphatidylcholine acyl-alkyl C38:4 | 0.82 (0.74-0.91 | 1.69E-04 |
| 26 | PC ae C40:1 | Phosphatidylcholine acyl-alkyl C40:1 | 0.78 (0.69-0.85) | 1.00E-06 |
| 27 | PC ae C40:3 | Phosphatidylcholine acyl-alkyl C40:3 | 0.73 (0.66-0.81) | 6.95E-09 |
| 28 | PC ae C40:4 | Phosphatidylcholine acyl-alkyl C40:4 | 0.71 (0.64-0.79) | 3.34E-10 |
| 29 | PC ae C42:0 | Phosphatidylcholine acyl-alkyl C42:0 | 0.72 (0.65-0.80) | 2.91E-09 |
| 30 | PC ae C42:1 | Phosphatidylcholine acyl-alkyl C42:1 | 0.67 (0.60-0.74) | 6.35E-13 |
| 31 | PC ae C42:3 | Phosphatidylcholine acyl-alkyl C42:3 | 0.78 (0.70-0.87) | 4.00E-06 |
| 32 | PC ae C42:4 | Phosphatidylcholine acyl-alkyl C42:4 | 0.63 (0.57-0.71) | 3.56E-16 |
| 33 | PC ae C42:5 | Phosphatidylcholine acyl-alkyl C42:5 | 0.83 (0.74-0.92) | 2.87E-04 |
| 34 | PC ae C44:4 | Phosphatidylcholine acyl-alkyl C44:4 | 0.64 (0.57-0.71) | 1.10E-15 |
| 35 | PC ae C44:6 | Phosphatidylcholine acyl-alkyl C44:6 | 0.61 (0.55-0.68) | 5.84E-18 |
| 36 | SM (OH) C14:1 | Hydroxysphingomyeline C14:1 | 0.68 (0.61-0.76) | 1.65E-11 |
| 37 | SM C16:0 | Sphingomyeline C16:0 | 0.64 (0.58-0.72) | 1.07E-14 |
| 38 | SM C16:1 | Sphingomyeline C16:1 | 0.63 (0.56-0.71) | 3.46E-14 |
| 39 | SM (OH) C16:1 | Hydroxysphingomyeline C16:1 | 0.75 (0.67-0.84) | 2.70E-07 |
| 40 | SM C18:1 | Sphingomyeline C18:1 | 0.72 (0.64-0.81) | 2.11E-08 |
| 41 | SM (OH) C22:2 | Hydroxysphingomyeline C22:2 | 0.57 (0.50-0.64) | 1.79E-19 |
| 42 | SM C24:1 | Sphingomyeline C24:1 | 0.68 (0.61-0.75) | 7.14E-13 |
| 43 | SM C26:1 | Sphingomyeline C26:1 | 0.81 (0.73-0.90) | 6.00E-05 |
| 44 | H1 | Hexose | 1.74 (1.55-1.95) | 4.28E-21 |

**b**

|  | Metabolites | | PD Vs. NGT (by HbA1c levels) | |
| --- | --- | --- | --- | --- |
| # | Abbreviation | Biochemical name | OR (95% CI), per SD | *P-*value |
| 1 | Ala | Alanine | 1.46 (1.30-1.64) | 1.13E-10 |
| 2 | Arg | Argine | 1.24 (1.16-1.38) | 8.40E-05 |
| 3 | Gln | Glutamine | 1.24 (1.11-1.40) | 2.19E-04 |
| 4 | Glu | Glutamate | 1.28 (1.15-1.43) | 1.00E-05 |
| 5 | Ile | Isoleucine | 1.43 (1.27-1.61) | 5.87E-09 |
| 6 | Leu | Leucine | 1.33 (1.18-1.50) | 2.00E-06 |
| 7 | Lys | Lysine | 1.34 (1.20-1.49) | 2.03E-07 |
| 8 | Met | Methionine | 1.35 (1.20-1.52) | 3.89E-07 |
| 9 | Phe | Phenylalanine | 1.35 (1.20-1.50) | 1.93E-07 |
| 10 | Pro | Proline | 1.26 (1.12-1.40) | 6.40E-05 |
| 11 | Tyr | Tyrosine | 1.29 (1.16-1.45) | 8.00E-06 |
| 12 | Val | Valine | 1.63 (1.44-1.84) | 3.49E-15 |
| 13 | lysoPC a C18:2 | lysoPhosphatidylcholine acyl C18:2 | 0.72 (0.64-0.80) | 1.29E-08 |
| 14 | PC aa C34:1 | Phosphatidylcholine diacyl C34:1 | 1.24 (1.12-1.39) | 8.20E-05 |
| 15 | PC aa C36:0 | Phosphatidylcholine diacyl C36:0 | 0.82 (0.73-0.91) | 1.83E-04 |
| 16 | PC aa C36:1 | Phosphatidylcholine diacyl C36:1 | 1.30 (1.17-1.45) | 2.00E-06 |
| 17 | PC aa C38:0 | Phosphatidylcholine diacyl C38:0 | 0.80 (0.72-0.89) | 5.30E-05 |
| 18 | PC aa C40:1 | Phosphatidylcholine diacyl C40:1 | 0.77 (0.69-0.86) | 4.00E-06 |
| 19 | PC aa C40:2 | Phosphatidylcholine diacyl C40:2 | 0.82 (0.74-0.92) | 3.66E-04 |
| 20 | PC aa C40:5 | Phosphatidylcholine diacyl C40:5 | 1.24 (1.11-1.38) | 1.18E-04 |
| 21 | PC aa C42:0 | Phosphatidylcholine diacyl C42:0 | 0.70 (0.62-0.78) | 3.85E-10 |
| 22 | PC aa C42:1 | Phosphatidylcholine diacyl C42:1 | 0.73 (0.65-0.81) | 2.28E-08 |
| 23 | PC ae C30:0 | Phosphatidylcholine acyl-alkyl C30:0 | 0.81 (0.73-0.91) | 1.70E-04 |
| 24 | PC ae C34:3 | Phosphatidylcholine acyl-alkyl C34:3 | 0.74 (0.67-0.83) | 9.99E-08 |
| 25 | PC ae C36:3 | Phosphatidylcholine acyl-alkyl C36:3 | 0.77 (0.69-0.85) | 1.00E-06 |
| 26 | PC ae C38:4 | Phosphatidylcholine acyl-alkyl C38:4 | 0.81 (0.73-0.90) | 9.30E-05 |
| 27 | PC ae C40:1 | Phosphatidylcholine acyl-alkyl C40:1 | 0.75 (0.68-0.84) | 3.63E-07 |
| 28 | PC ae C40:3 | Phosphatidylcholine acyl-alkyl C40:3 | 0.79 (0.71-0.88) | 1.70E-05 |
| 29 | PC ae C40:4 | Phosphatidylcholine acyl-alkyl C40:4 | 0.73 (0.65-0.81) | 1.70E-08 |
| 30 | PC ae C42:1 | Phosphatidylcholine acyl-alkyl C42:1 | 0.67 (0.60-0.75) | 1.34E-11 |
| 31 | PC ae C42:3 | Phosphatidylcholine acyl-alkyl C42:3 | 0.76 (0.68-0.85) | 1.00E-06 |
| 32 | PC ae C42:4 | Phosphatidylcholine acyl-alkyl C42:4 | 0.71 (0.64-0.80) | 1.93E-09 |
| 33 | PC ae C44:4 | Phosphatidylcholine acyl-alkyl C44:4 | 0.74 (0.67-0.83) | 1.47E-07 |
| 34 | PC ae C44:5 | Phosphatidylcholine acyl-alkyl C44:5 | 0.82 (0.74-0.92) | 3.24E-04 |
| 35 | PC ae C44:6 | Phosphatidylcholine acyl-alkyl C44:6 | 0.69 (0.62-0.77) | 7.63E-11 |
| 36 | SM C16:0 | Sphingomyeline C16:0 | 0.81 (0.72-0.90) | 1.70E-04 |
| 37 | SM C16:1 | Sphingomyeline C16:1 | 0.79 (0.70-0.89) | 8.90E-05 |
| 38 | SM (OH) C22:2 | Hydroxysphingomyeline C22:2 | 0.71 (0.63-0.80) | 1.47E-08 |
| 39 | SM C24:1 | Sphingomyeline C24:1 | 0.75 (0.67-0.83) | 1.19E-07 |

**c**

|  | Metabolites | | IFG Vs. NGT | |
| --- | --- | --- | --- | --- |
| # | Abbreviation | Biochemical name | OR (95% CI), per SD | *P*-value |
| 1 | C18:1 | Octadecenoylcarnitine | 1.55 (1.34-1.80) | 7.32E-09 |
| 2 | Ala | Alanine | 1.50 (1.28-1.75) | 1.00E-06 |
| 3 | Glu | Glutamate | 1.34 (1.15-1.56) | 1.27E-04 |
| 4 | Gly | Glycine | 0.66 (0.55-0.78) | 1.00E-06 |
| 5 | Val | Valine | 1.37 (1.16-1.61) | 2.01E-04 |
| 6 | Creatinine | Creatinine | 0.51 (0.42-0.63) | 1.19E-10 |
| 7 | Kynurenine | Kynurenine | 0.65 (0.55-0.76) | 1.68E-07 |
| 8 | Taurine | Taurine | 1.35 (1.16-1.58) | 1.27E-04 |
| 9 | lysoPC a C16:0 | lysoPhosphatidylcholine acyl C16:0 | 1.38 (1.18-1.61) | 4.90E-05 |
| 10 | lysoPC a C16:1 | lysoPhosphatidylcholine acyl C16:1 | 1.30 (1.13-1.51) | 2.99E-04 |
| 11 | lysoPC a C18:0 | lysoPhosphatidylcholine acyl C18:0 | 1.34 (1.14-1.56) | 2.63E-04 |
| 12 | lysoPC a C20:4 | lysoPhosphatidylcholine acyl C20:4 | 1.37 (1.17-1.61) | 1.04E-04 |
| 13 | PC aa C34:4 | Phosphatidylcholine diacyl C34:4 | 1.41 (1.22-1.63) | 5.00E-06 |
| 14 | PC aa C36:5 | Phosphatidylcholine diacyl C36:5 | 1.44 (1.24-1.68) | 1.00E-06 |
| 15 | PC aa C36:6 | Phosphatidylcholine diacyl C36:6 | 1.31 (1.13-1.51) | 3.81E-04 |
| 16 | PC aa C38:5 | Phosphatidylcholine diacyl C38:5 | 1.43 (1.23-1.66) | 2.00E-06 |
| 17 | PC aa C42:0 | Phosphatidylcholine diacyl C42:0 | 0.68 (0.59-0.80) | 1.00E-06 |
| 18 | PC aa C42:1 | Phosphatidylcholine diacyl C42:1 | 0.69 (0.59-0.80) | 2.00E-06 |
| 19 | PC ae C30:0 | Phosphatidylcholine acyl-alkyl C30:0 | 0.67 (0.57-0.78) | 2.74E-07 |
| 20 | PC ae C34:3 | Phosphatidylcholine acyl-alkyl C34:3 | 0.68 (0.58-0.79) | 1.00E-06 |
| 21 | PC ae C36:3 | Phosphatidylcholine acyl-alkyl C36:3 | 0.73 (0.63-0.85) | 6.10E-05 |
| 22 | PC ae C40:3 | Phosphatidylcholine acyl-alkyl C40:3 | 0.73 (0.62-0.85) | 4.30E-05 |
| 23 | PC ae C40:4 | Phosphatidylcholine acyl-alkyl C40:4 | 0.74 (0.64-0.86) | 9.10E-05 |
| 24 | PC ae C42:4 | Phosphatidylcholine acyl-alkyl C42:4 | 0.63 (0.54-0.74) | 9.55E-09 |
| 25 | PC ae C44:4 | Phosphatidylcholine acyl-alkyl C44:4 | 0.58 (0.50-0.68) | 2.07E-11 |
| 26 | PC ae C44:6 | Phosphatidylcholine acyl-alkyl C44:6 | 0.62 (0.53-0.72) | 1.35E-09 |
| 27 | SM (OH) C14:1 | Hydroxysphingomyeline C14:1 | 0.64 (0.54-0.75) | 2.42E-08 |
| 28 | SM C16:0 | Sphingomyeline C16:0 | 0.64 (0.55-0.75) | 2.05E-08 |
| 29 | SM C16:1 | Sphingomyeline C16:1 | 0.58 (0.49-0.69) | 3.72E-10 |
| 30 | SM (OH) C16:1 | Hydroxysphingomyeline C16:1 | 0.70 (0.60-0.81) | 4.00E-06 |
| 31 | SM C18:1 | Sphingomyeline C18:1 | 0.59 (0.50-0.70) | 5.17E-10 |
| 32 | SM (OH) C22:2 | Hydroxysphingomyeline C22:2 | 0.55 (0.46-0.65) | 9.75E-12 |
| 33 | SM C24:1 | Sphingomyeline C24:1 | 0.73 (0.63-0.84) | 1.90E-05 |
| 34 | H1 | Hexose | 3.09 (2.56-3.74) | 1.61E-31 |

**d**

|  | Metabolites | | IGT Vs. NGT | |
| --- | --- | --- | --- | --- |
| # | Abbreviation | Biochemical name | OR (95% CI), per SD | *P*-value |
| 1 | Ala | Alanine | 1.41 (1.21-1.65) | 1.50E-05 |
| 2 | Arg | Arginine | 1.42 (1.21-1.66) | 1.20E-05 |
| 3 | Gly | Glycine | 0.73 (0.62-0.85) | 4.30E-05 |
| 4 | Ile | Isoleucine | 1.37 (1.16-1.60) | 1.51E-04 |
| 5 | Met | Methionine | 1.32 (1.14-1.54) | 3.52E-04 |
| 6 | Phe | Phenylalanine | 1.48 (1.27-1.72) | 4.35E-07 |
| 7 | Val | Valine | 1.69 (1.44-1.99) | 3.24E-10 |
| 8 | lysoPC a C18:2 | lysoPhosphatidylcholine acyl C18:2 | 0.67 (0.57-0.78) | 4.70E-07 |
| 9 | PC aa C36:1 | Phosphatidylcholine diacyl C36:1 | 1.36 (1.17-1.57) | 4.10E-05 |
| 10 | PC aa C40:6 | Phosphatidylcholine diacyl C40:6 | 1.33 (1.15-1.54) | 1.58E-04 |
| 11 | PC aa C42:0 | Phosphatidylcholine diacyl C42:0 | 0.65 (0.55-0.75) | 3.20E-08 |
| 12 | PC aa C42:1 | Phosphatidylcholine diacyl C42:1 | 0.72 (0.62-0.84) | 2.40E-05 |
| 13 | PC ae C34:2 | Phosphatidylcholine acyl-alkyl C34:2 | 0.75 (0.64-0.87) | 1.50E-04 |
| 14 | PC ae C34:3 | Phosphatidylcholine acyl-alkyl C34:3 | 0.64 (0.55-0.75) | 2.46E-08 |
| 15 | PC ae C36:3 | Phosphatidylcholine acyl-alkyl C36:3 | 0.66 (0.57-0.77) | 1.14E-07 |
| 16 | PC ae C36:4 | Phosphatidylcholine acyl-alkyl C36:4 | 0.77 (0.66-0.89) | 3.55E-04 |
| 17 | PC ae C38:4 | Phosphatidylcholine acyl-alkyl C38:4 | 0.75 (0.65-0.87) | 8.30E-05 |
| 18 | PC ae C40:1 | Phosphatidylcholine acyl-alkyl C40:1 | 0.75 (0.64-0.87) | 1.20E-04 |
| 19 | PC ae C40:3 | Phosphatidylcholine acyl-alkyl C40:3 | 0.72 (0.62-0.84) | 2.00E-05 |
| 20 | PC ae C40:4 | Phosphatidylcholine acyl-alkyl C40:4 | 0.64 (0.55-0.75) | 1.15E-08 |
| 21 | PC ae C42:1 | Phosphatidylcholine acyl-alkyl C42:1 | 0.65 (0.55-0.75) | 3.82E-08 |
| 22 | PC ae C42:3 | Phosphatidylcholine acyl-alkyl C42:3 | 0.73 (0.63-0.84) | 2.60E-05 |
| 23 | PC ae C42:4 | Phosphatidylcholine acyl-alkyl C42:4 | 0.63 (0.54-0.73) | 2.51E-09 |
| 24 | PC ae C44:4 | Phosphatidylcholine acyl-alkyl C44:4 | 0.69 (0.59-0.80) | 2.00E-06 |
| 25 | PC ae C44:5 | Phosphatidylcholine acyl-alkyl C44:5 | 0.77 (0.66-0.88) | 2.65E-04 |
| 26 | PC ae C44:6 | Phosphatidylcholine acyl-alkyl C44:6 | 0.64 (0.55-0.74) | 9.96E-09 |
| 27 | SM C16:0 | Sphingomyeline C16:0 | 0.68 (0.58-0.79) | 1.00E-06 |
| 28 | SM (OH) C22:2 | Hydroxysphingomyeline C22:2 | 0.69 (0.58-0.82) | 2.60E-05 |
| 29 | SM C24:1 | Sphingomyeline C24:1 | 0.66 (0.57-0.77) | 5.78E-08 |

**e**

|  | Metabolites | | IFG+IGT Vs. NGT | |
| --- | --- | --- | --- | --- |
| # | Abbreviation | Biochemical name | OR (95% CI), per SD | *P*-value |
| 1 | C18:1 | Octadecenoylcarnitine | 1.38 (1.16-1.63) | 1.84E-04 |
| 2 | Ala | Alanine | 1.70 (1.42-2.04) | 5.92E-09 |
| 3 | Gly | Glycine | 0.66 (0.55-0.79) | 4.00E-06 |
| 4 | Val | Valine | 1.51 (1.26-1.81) | 9.00E-06 |
| 5 | Kynurenine | Kynurenine | 0.64 (0.54-0.77) | 4.53E-07 |
| 6 | lysoPC a C18:2 | lysoPhosphatidylcholine acyl C18:2 | 0.64 (0.54-0.76) | 1.00E-06 |
| 7 | PC aa C34:4 | Phosphatidylcholine diacyl C34:4 | 1.38 (1.18-1.63) | 9.40E-05 |
| 8 | PC aa C36:5 | Phosphatidylcholine diacyl C36:5 | 1.60 (1.35-1.91) | 8.57E-08 |
| 9 | PC aa C36:6 | Phosphatidylcholine diacyl C36:6 | 1.44 (1.22-1.71) | 1.90E-05 |
| 10 | PC aa C38:5 | Phosphatidylcholine diacyl C38:5 | 1.52 (1.28-1.80) | 1.00E-06 |
| 11 | PC aa C40:5 | Phosphatidylcholine diacyl C40:5 | 1.42 (1.20-1.69) | 5.70E-05 |
| 12 | PC aa C42:0 | Phosphatidylcholine diacyl C42:0 | 0.70 (0.59-0.83) | 3.10E-05 |
| 13 | PC aa C42:1 | Phosphatidylcholine diacyl C42:1 | 0.68 (0.58-0.81) | 9.00E-06 |
| 14 | PC ae C34:2 | Phosphatidylcholine acyl-alkyl C34:2 | 0.64 (0.54-0.76) | 2.32E-07 |
| 15 | PC ae C34:3 | Phosphatidylcholine acyl-alkyl C34:3 | 0.53 (0.45-0.63) | 1.12E-12 |
| 16 | PC ae C36:3 | Phosphatidylcholine acyl-alkyl C36:3 | 0.64 (0.54-0.76) | 2.92E-07 |
| 17 | PC ae C40:3 | Phosphatidylcholine acyl-alkyl C40:3 | 0.71 (0.60-0.84) | 6.90E-05 |
| 18 | PC ae C42:1 | Phosphatidylcholine acyl-alkyl C42:1 | 0.70 (0.59-0.84) | 7.00E-05 |
| 19 | PC ae C42:4 | Phosphatidylcholine acyl-alkyl C42:4 | 0.65 (0.55-0.77) | 4.81E-07 |
| 20 | PC ae C44:4 | Phosphatidylcholine acyl-alkyl C44:4 | 0.67 (0.58-0.79) | 3.00E-06 |
| 21 | PC ae C44:6 | Phosphatidylcholine acyl-alkyl C44:6 | 0.66 (0.56-0.78) | 2.00E-06 |
| 22 | SM (OH) C14:1 | Hydroxysphingomyeline C14:1 | 0.63 (0.53-0.75) | 1.29E-07 |
| 23 | SM C16:0 | Sphingomyeline C16:0 | 0.57 (0.48-0.67) | 1.27E-10 |
| 24 | SM C16:1 | Sphingomyeline C16:1 | 0.54 (0.45-0.65) | 2.44E-11 |
| 25 | SM C18:1 | Sphingomyeline C18:1 | 0.65 (0.54-0.78) | 3.00E-06 |
| 26 | SM (OH) C22:2 | Hydroxysphingomyeline C22:2 | 0.55 (0.45-0.66) | 2.71E-10 |
| 27 | SM C24:1 | Sphingomyeline C24:1 | 0.70 (0.60-0.83) | 2.00E-05 |
| 28 | H1 | Hexose | 3.25 (2.64-3.99) | 2.39E-29 |

**f**

|  | Metabolites | | Q4 Vs. Q1 | |
| --- | --- | --- | --- | --- |
| # | Abbreviation | Biochemical name | OR (95% CI), per SD | *p*-value |
| 1 | Ala | Alanine | 2.22 (1.83-2.70) | 8.37E-16 |
| 2 | Arg | Argine | 1.67 (1.40-1.99) | 7.91E-09 |
| 3 | Glu | Glutamate | 1.41 (1.18-1.69) | 1.95E-04 |
| 4 | Gly | Glycine | 0.73 (0.61-0.87) | 4.03E-04 |
| 5 | Ile | Isoleucine | 1.48 (1.22-1.79) | 6.10E-05 |
| 6 | Pro | Proline | 1.69 (1.41-2.03) | 2.29E-08 |
| 7 | Tyr | Tyrosine | 1.55 (1.29-1.87) | 2.00E-06 |
| 8 | Val | Valine | 1.83 (1.50-2.22) | 2.15E-09 |
| 9 | PC aa C32:1 | Phosphatidylcholine diacyl C32:1 | 1.41 (1.18-1.68) | 1.28E-04 |
| 10 | PC aa C36:1 | Phosphatidylcholine diacyl C36:1 | 1.41 (1.19-1.68) | 9.50E-05 |
| 11 | PC aa C42:5 | Phosphatidylcholine diacyl C42:5 | 1.41 (1.20-1.67) | 5.20E-05 |
| 12 | PC ae C34:3 | Phosphatidylcholine acyl-alkyl C34:3 | 0.56 (0.46-0.67) | 2.56E-10 |
| 13 | PC ae C36:3 | Phosphatidylcholine acyl-alkyl C36:3 | 0.72 (0.60-0.85) | 1.76E-04 |
| 14 | PC ae C40:3 | Phosphatidylcholine acyl-alkyl C40:3 | 0.70 (0.59-0.83) | 4.90E-05 |
| 15 | PC ae C40:4 | Phosphatidylcholine acyl-alkyl C40:4 | 0.71 (0.60-0.84) | 7.20E-05 |
| 16 | PC ae C42:1 | Phosphatidylcholine acyl-alkyl C42:1 | 0.62 (0.52-0.75) | 2.29E-07 |
| 17 | PC ae C42:4 | Phosphatidylcholine acyl-alkyl C42:4 | 0.65 (0.55-0.78) | 2.00E-06 |
| 18 | PC ae C44:4 | Phosphatidylcholine acyl-alkyl C44:5 | 0.64 (0.53-0.76) | 1.00E-06 |
| 19 | PC ae C44:6 | Phosphatidylcholine acyl-alkyl C44:6 | 0.71 (0.60-0.85) | 1.58E-04 |
| 20 | SM C16:0 | Sphingomyeline C16:0 | 0.63 (0.52-0.75) | 4.05E-07 |
| 21 | SM C16:1 | Sphingomyeline C16:1 | 0.64 (0.53-0.77) | 3.00E-06 |
| 22 | SM (OH) C16:1 | Hydroxysphingomyeline C16:1 | 0.71 (0.59-0.84) | 1.26E-04 |
| 23 | SM (OH) C22:2 | Hydroxysphingomyeline C22:2 | 0.58 (0.48-0.71) | 4.25E-08 |
| 24 | SM C24:1 | Sphingomyeline C24:1 | 0.73 (0.62-0.86) | 2.00E-04 |
| 25 | H1 | Hexose | 2.5 (2.06-3.07) | 2.62E-19 |

**Supplementary Table S3.** Significant metabolites in response to elevated levels of glucose, HbA1c, and HOMA-IR levels. (a) Multivariable linear regression analysis between metabolites and fasting glucose values in the KARE S2 baseline (n=1,723). 47 metabolites showed significant concentration differences corresponding to the fasting glucose increment in the analysis with adjustment for age, sex and BMI. The Bonferroni correction was applied for multiple tests and *P* < 4.07E-04 was considered to be statistically significant difference. (b) Multivariable linear regression analysis between metabolites and 2-h glucose values in the KARE S2 baseline (n=1,723). 52 metabolites showed significant concentration differences corresponding to the 2-h PPG increment. (c) Multivariable linear regression analysis between metabolites and HbAlc values in the KARE S2 baseline (n=1,723). 47 metabolites showed significant concentration differences corresponding to the elevated HbA1c level. (d) Multivariable linear regression analysis between metabolites and HOMA-IR values in the KARE S2 baseline (n=1,723). 37 metabolites showed significant concentration differences corresponding to the HOMA-IR level.

**a**

|  | Metabolites | | Fasting glucose | |
| --- | --- | --- | --- | --- |
| # | Abbreviation | Biochemical name | Beta (95% CI) | *P*-value |
| 1 | C18:1 | Octadecenoylcarnitine | 0.137 (0.090, 0.182) | 6.93E-09 |
| 2 | Ala | Alanine | 0.221 (0.177, 0.266) | 6.23E-22 |
| 3 | Glu | Glutamate | 0.112 (0.067, 0.157) | 1.00E-06 |
| 4 | Gly | Glycine | -0.126 (-0.171, -0.080) | 6.51E-08 |
| 5 | Phe | Phenylalanine | 0.100 (0.054, 0.146) | 2.10E-05 |
| 6 | Pro | Proline | 0.143 (0.097, 0.188) | 1.39E-09 |
| 7 | Tyr | Tyrosine | 0.128 (0.082, 0.174) | 6.52E-08 |
| 8 | Val | Valine | 0.190 (0.143, 0.238) | 6.64E-15 |
| 9 | Creatinine | Creatinine | -0.119 (-0.175, -0.069) | 8.00E-06 |
| 10 | Kynurenine | Kynurenine | -0.105 (-0.150, -0.059) | 6.00E-06 |
| 11 | Putrescine | Putrescine | -0.080 (-0.124, -0.036) | 3.43E-04 |
| 12 | lysoPC a C16:0 | lysoPhosphatidylcholine acyl C16:0 | 0.111 (0.066, 0.156) | 1.00E-06 |
| 13 | lysoPC a C16:1 | lysoPhosphatidylcholine acyl C16:1 | 0.095 (0.050, 0.140) | 3.60E-05 |
| 14 | lysoPC a C18:0 | lysoPhosphatidylcholine acyl C18:0 | 0.086 (0.042, 0.130) | 1.29E-04 |
| 15 | lysoPC a C18:2 | lysoPhosphatidylcholine acyl C18:2 | -0.104 (-0.150, -0.057) | 1.20E-05 |
| 16 | lysoPC a C20:4 | lysoPhosphatidylcholine acyl C20:4 | 0.123 (0.078, 0.169) | 1.34E-07 |
| 17 | PC aa C34:1 | Phosphatidylcholine diacyl C34:1 | 0.085 (0.040, 0.130) | 2.46E-04 |
| 18 | PC aa C34:4 | Phosphatidylcholine diacyl C34:4 | 0.129 (0.085, 0.172) | 1.10E-08 |
| 19 | PC aa C36:1 | Phosphatidylcholine diacyl C36:1 | 0.084 (0.039, 0.129) | 2.51E-04 |
| 20 | PC aa C36:5 | Phosphatidylcholine diacyl C36:5 | 0.162 (0.118, 0.205) | 6.68E-13 |
| 21 | PC aa C36:6 | Phosphatidylcholine diacyl C36:6 | 0.114 (0.069, 0.158) | 1.00E-06 |
| 22 | PC aa C38:5 | Phosphatidylcholine diacyl C38:5 | 0.155 (0.111, 0.199) | 4.89E-12 |
| 23 | PC aa C40:5 | Phosphatidylcholine diacyl C40:5 | 0.121 (0.076, 0.166) | 1.41E-07 |
| 24 | PC aa C42:0 | Phosphatidylcholine diacyl C42:0 | -0.135 (-0.181, -0.090) | 5.78E-09 |
| 25 | PC aa C42:1 | Phosphatidylcholine diacyl C42:1 | -0.132 (-0.177, -0.086) | 1.40E-08 |
| 26 | PC aa C42:5 | Phosphatidylcholine diacyl C42:5 | 0.114 (0.070, 0.158) | 3.27E-07 |
| 27 | PC ae C30:0 | Phosphatidylcholine acyl-alkyl C30:0 | -0.127 (-0.171, -0.082) | 2.47E-08 |
| 28 | PC ae C34:2 | Phosphatidylcholine acyl-alkyl C34:2 | -0.116 (-0.160, -0.072) | 3.11E-07 |
| 29 | PC ae C34:3 | Phosphatidylcholine acyl-alkyl C34:3 | -0.191 (-0.235, -0.147) | 3.11E-17 |
| 30 | PC ae C36:3 | Phosphatidylcholine acyl-alkyl C36:3 | -0.140 (-0.184, -0.096) | 6.11E-10 |
| 31 | PC ae C38:3 | Phosphatidylcholine acyl-alkyl C38:3 | -0.092 (-0.137, -0.048) | 4.90E-05 |
| 32 | PC ae C40:1 | Phosphatidylcholine acyl-alkyl C40:1 | -0.093 (-0.138, -0.049) | 4.60E-05 |
| 33 | PC ae C40:3 | Phosphatidylcholine acyl-alkyl C40:3 | -0.117 (-0.162, -0.073) | 2.80E-07 |
| 34 | PC ae C40:4 | Phosphatidylcholine acyl-alkyl C40:4 | -0.100 (-0.144, -0.056) | 1.00E-05 |
| 35 | PC ae C40:5 | Phosphatidylcholine acyl-alkyl C40:5 | 0.091 (0.046, 0.135) | 6.80E-05 |
| 36 | PC ae C42:1 | Phosphatidylcholine acyl-alkyl C42:1 | -0.136 (-0.181, -0.090) | 5.68E-09 |
| 37 | PC ae C42:4 | Phosphatidylcholine acyl-alkyl C42:4 | -0.167 (-0.211, -0.122) | 2.71E-13 |
| 38 | PC ae C44:4 | Phosphatidylcholine acyl-alkyl C44:4 | -0.180 (-0.224, -0.135) | 4.05E-15 |
| 39 | PC ae C44:6 | Phosphatidylcholine acyl-alkyl C44:6 | -0.159 (-0.204, -0.114) | 6.49E-12 |
| 40 | SM (OH) C14:1 | Hydroxysphingomyeline C14:1 | -0.180 (-0.226, -0.134) | 3.41E-14 |
| 41 | SM C16:0 | Sphingomyeline C16:0 | -0.188 (-0.233, -0.143) | 5.33E-16 |
| 42 | SM C16:1 | Sphingomyeline C16:1 | -0.215 (-0.263, -0.167) | 3.54E-18 |
| 43 | SM C18:1 | Sphingomyeline C18:1 | -0.187 (-0.235, -0.139) | 3.21E-14 |
| 44 | SM (OH) C16:1 | Hydroxysphingomyeline C16:1 | -0.150 (-0.196, -0.104) | 1.59E-10 |
| 45 | SM (OH) C22:2 | Hydroxysphingomyeline C22:2 | -0.229 (-0.277, -0.181) | 2.99E-20 |
| 46 | SM C24:1 | Sphingomyeline C24:1 | -0.124 (-0.168, -0.080) | 3.28E-08 |
| 47 | H1 | Hexose | 0.500 (0.461, 0.538) | 4.45E-120 |

**b**

|  | Metabolites | | 2-h PPG | |
| --- | --- | --- | --- | --- |
| # | Abbreviation | Biochemical name | Beta (95% CI) | *P*-value |
| 1 | C16 | Hexadecanoylcarnitine | 0.091 (0.045, 0.138) | 1.36E-04 |
| 2 | C18:1 | Octadecenoylcarnitine | 0.104 (0.057, 0.151) | 1.50E-05 |
| 3 | Ala | Alanine | 0.177 (0.131, 0.223) | 5.32E-14 |
| 4 | Cit | Citrulline | -0.108 (-0.155, -0.061) | 7.00E-06 |
| 5 | Gly | Glycine | -0.141 (-0.187, -0.095) | 2.41E-09 |
| 6 | Ile | Isoleucine | 0.090 (0.040, 0.139) | 4.07E-04 |
| 7 | Lys | Lysine | 0.086 (0.040, 0.131) | 2.31E-04 |
| 8 | Met | Methionine | 0.100 (0.053, 0.148) | 4.10E-05 |
| 9 | Phe | Phenylalanine | 0.125 (0.078, 0.171) | 1.68E-07 |
| 10 | Pro | Proline | 0.104 (0.057, 0.151) | 1.50E-05 |
| 11 | Val | Valine | 0.217 (0.169, 0.266) | 2.00E-18 |
| 12 | Kynrenine | Kynrenine | -0.085 (-0.131, -0.039) | 2.95E-04 |
| 13 | Putrescine | Putrescine | -0.098 (-0.143, -0.054) | 1.60E-05 |
| 14 | lysoPC a C18:1 | lysoPhosphatidylcholine acyl C18:1 | -0.104 (-0.151, -0.058) | 1.20E-05 |
| 15 | lysoPC a C18:2 | lysoPhosphatidylcholine acyl C18:2 | -0.196 (-0.243, -0.149) | 3.58E-16 |
| 16 | PC aa C34:3 | Phosphatidylcholine diacyl C34:3 | -0.103 (-0.149, -0.057) | 1.10E-05 |
| 17 | PC aa C36:0 | Phosphatidylcholine diacyl C36:0 | -0.085 (-0.130 (-0.039) | 2.56E-04 |
| 18 | PC aa C38:0 | Phosphatidylcholine diacyl C38:0 | -0.084 (-0.130, -0.039) | 2.79E-04 |
| 19 | PC aa C38:6 | Phosphatidylcholine diacyl C38:6 | 0.095 (0.049, 0.140) | 4.60E-05 |
| 20 | PC aa C40:1 | Phosphatidylcholine diacyl C40:1 | -0.094 (-0.140, -0.049) | 5.00E-05 |
| 21 | PC aa C40:2 | Phosphatidylcholine diacyl C40:2 | -0.092 (-0.137, -0.047) | 5.80E-05 |
| 22 | PC aa C40:3 | Phosphatidylcholine diacyl C40:3 | -0.097 (-0.142, -0.052) | 2.30E-05 |
| 23 | PC aa C40:5 | Phosphatidylcholine diacyl C40:5 | 0.115 (0.070, 0.161) | 1.00E-06 |
| 24 | PC aa C40:6 | Phosphatidylcholine diacyl C40:6 | 0.109 (0.064, 0.154) | 2.00E-06 |
| 25 | PC aa C42:0 | Phosphatidylcholine diacyl C42:0 | -0.129 (-0.175, -0.082) | 5.80E-08 |
| 26 | PC aa C42:1 | Phosphatidylcholine diacyl C42:1 | -0.104 (-0.150, -0.057) | 1.20E-05 |
| 27 | PC aa C42:4 | Phosphatidylcholine diacyl C42:4 | -0.091 (-0.136, -0.047) | 6.70E-05 |
| 28 | PC ae C30:0 | Phosphatidylcholine acyl-alkyl C30:0 | -0.094 (-0.139, -0.048) | 5.50E-05 |
| 29 | PC ae C32:1 | Phosphatidylcholine acyl-alkyl C32:1 | -0.105 (-0.149, -0.060) | 4.00E-06 |
| 30 | PC ae C32:2 | Phosphatidylcholine acyl-alkyl C32:2 | -0.090 (-0.135, -0.045) | 8.50E-05 |
| 31 | PC ae C34:1 | Phosphatidylcholine acyl-alkyl C34:1 | -0.105 (-0.151, -0.059) | 7.00E-06 |
| 32 | PC ae C34:2 | Phosphatidylcholine acyl-alkyl C34:2 | -0.152 (-0.197, -0.107) | 3.54E-11 |
| 33 | PC ae C34:3 | Phosphatidylcholine acyl-alkyl C34:3 | -0.227 (-0.271, -0.182) | 5.79E-23 |
| 34 | PC ae C36:2 | Phosphatidylcholine acyl-alkyl C36:2 | -0.111 (-0.156, -0.066) | 1.00E-06 |
| 35 | PC ae C36:3 | Phosphatidylcholine acyl-alkyl C36:3 | -0.194 (-0.238, -0.149) | 2.61E-17 |
| 36 | PC ae C36:4 | Phosphatidylcholine acyl-alkyl C36:4 | -0.085 (-0.130, -0.040) | 1.94E-04 |
| 37 | PC ae C38:4 | Phosphatidylcholine acyl-alkyl C38:4 | -0.093 (-0.138, -0.048) | 4.60E-05 |
| 38 | PC ae C40:1 | Phosphatidylcholine acyl-alkyl C40:1 | -0.134 (-0.179, -0.089) | 8.76E-09 |
| 39 | PC ae C40:3 | Phosphatidylcholine acyl-alkyl C40:3 | -0.127 (-0.173, -0.082) | 3.87E-08 |
| 40 | PC ae C40:4 | Phosphatidylcholine acyl-alkyl C40:4 | -0.139 (-0.184, -0.094) | 1.55E-09 |
| 41 | PC ae C42:1 | Phosphatidylcholine acyl-alkyl C42:1 | -0.177 (-0.223, -0.131) | 6.94E-14 |
| 42 | PC ae C42:2 | Phosphatidylcholine acyl-alkyl C42:2 | -0.085 (-0.131, -0.040) | 2.06E-04 |
| 43 | PC ae C42:3 | Phosphatidylcholine acyl-alkyl C42:3 | -0.110 (-0.156, -0.065) | 2.00E-06 |
| 44 | PC ae C42:4 | Phosphatidylcholine acyl-alkyl C42:4 | -0.156 (-0.202, -0.111) | 1.91E-11 |
| 45 | PC ae C44:4 | Phosphatidylcholine acyl-alkyl C44:4 | -0.117 (-0.163, -0.071) | 1.00E-06 |
| 46 | PC ae C44:6 | Phosphatidylcholine acyl-alkyl C44:6 | -0.133 (-0.179, -0.087) | 1.84E-08 |
| 47 | SM (OH) C14:1 | Hydroxysphingomyeline C14:1 | -0.099 (-0.147, -0.052) | 4.40E-05 |
| 48 | SM C16:0 | Sphingomyeline C16:0 | -0.179 (-0.225, -0.133) | 4.16E-14 |
| 49 | SM C16:1 | Sphingomyeline C16:1 | -0.149 (-0.199, -0.100) | 3.55E-09 |
| 50 | SM (OH) C22:2 | Hydroxysphingomyeline C22:2 | -0.141 (-0.192, -0.092) | 2.82E-08 |
| 51 | SM C24:1 | Sphingomyeline C24:1 | -0.148 (-0.193, -0.104) | 8.41E-11 |
| 52 | H1 | Hexose | 0.131 (0.085, 0.177) | 2.60E-08 |

**c**

|  | | Metabolites | | HbA1c | |
| --- | --- | --- | --- | --- | --- |
| # | | Abbreviation | Biochemical name | Beta (95% CI) | *P*-value |
| 1 | Ala | Alanine | 0.073 (0.055, 0.090) | 1.53E-15 |  |
| 2 | Arg | Arginine | 0.045 (0.027, 0.062) | 4.25E-07 |  |
| 3 | Glu | Glutamate | 0.054 (0.036, 0.072) | 2.77E-09 |  |
| 4 | Gly | Glycine | -0.033 (-0.051, -0.015) | 2.73E-04 |  |
| 5 | Ile | Isoleucine | 0.075 (0.056, 0.094) | 1.08E-14 |  |
| 6 | Leu | Leucine | 0.064 (0.046, 0.083) | 2.84E-11 |  |
| 7 | Lys | Lysine | 0.054 (0.036, 0.072) | 1.92E-09 |  |
| 8 | Met | Methionine | 0.054 (0.036, 0.073 | 1.04E-08 |  |
| 9 | Orn | Ornithine | 0.033 (0.015, 0.050) | 2.70E-04 |  |
| 10 | Phe | Phenylalanine | 0.051 (0.033, 0.069) | 2.92E-08 |  |
| 11 | Pro | Proline | 0.041 (0.023, 0.059) | 1.10E-05 |  |
| 12 | Tyr | Tyrosine | 0.045 (0.026, 0.063) | 2.00E-06 |  |
| 13 | Val | Valine | 0.088 (0.070, 0.107) | 3.41E-20 |  |
| 14 | Taurine | Taurine | 0.043 (0.025, 0.060) | 1.00E-06 |  |
| 15 | lysoPC a C17:0 | lysoPhosphatidylcholine acyl C17:0 | -0.033 (-0.050, -0.015) | 2.10E-04 |  |
| 16 | lysoPC a C18:2 | lysoPhosphatidylcholine acyl C18:2 | -0.049 (-0.067, -0.031) | 1.81E-07 |  |
| 17 | PC aa C32:1 | Phosphatidylcholine diacyl C32:1 | 0.035 (0.017, 0.053) | 1.62E-04 |  |
| 18 | PC aa C34:1 | Phosphatidylcholine diacyl C34:1 | 0.039 (0.021, 0.057) | 1.70E-05 |  |
| 19 | PC aa C36:0 | Phosphatidylcholine diacyl C36:0 | -0.036 (-0.053, -0.018) | 6.20E-05 |  |
| 20 | PC aa C36:1 | Phosphatidylcholine diacyl C36:1 | 0.049 (0.032, 0.067) | 4.68E-08 |  |
| 21 | PC aa C38:0 | Phosphatidylcholine diacyl C38:0 | -0.042 (-0.060, -0.025) | 3.00E-06 |  |
| 22 | PC aa C40:1 | Phosphatidylcholine diacyl C40:1 | -0.048 (-0.066, -0.030) | 8.72E-08 |  |
| 23 | PC aa C40:2 | Phosphatidylcholine diacyl C40:2 | -0.036 (-0.054, -0.019) | 4.10E-05 |  |
| 24 | PC aa C40:5 | Phosphatidylcholine diacyl C40:5 | 0.045 (0.027, 0.063) | 1.00E-06 |  |
| 25 | PC aa C42:0 | Phosphatidylcholine diacyl C42:0 | -0.061 (-0.079, -0.043) | 2.64E-11 |  |
| 26 | PC aa C42:1 | Phosphatidylcholine diacyl C42:1 | -0.058 (-0.076, -0.041) | 1.80E-10 |  |
| 27 | PC aa C42:4 | Phosphatidylcholine diacyl C42:4 | -0.044 (-0.061, -0.027) | 1.00E-06 |  |
| 28 | PC ae C30:0 | Phosphatidylcholine acyl-alkyl C30:0 | -0.039 (-0.057, -0.021) | 1.50E-05 |  |
| 29 | PC ae C34:3 | Phosphatidylcholine acyl-alkyl C34:3 | -0.054 (-0.072, -0.037) | 1.60E-09 |  |
| 30 | PC ae C36:3 | Phosphatidylcholine acyl-alkyl C36:3 | -0.047 (-0.064, -0.029) | 1.82E-07 |  |
| 31 | PC ae C38:4 | Phosphatidylcholine acyl-alkyl C38:4 | -0.034 (-0.051, -0.017) | 1.29E-04 |  |
| 32 | PC ae C40:1 | Phosphatidylcholine acyl-alkyl C40:1 | -0.042 (-0.059, -0.024) | 4.00E-06 |  |
| 33 | PC ae C40:3 | Phosphatidylcholine acyl-alkyl C40:3 | -0.045 (-0.062, -0.027) | 1.00E-06 |  |
| 34 | PC ae C40:4 | Phosphatidylcholine acyl-alkyl C40:4 | -0.055 (-0.073, -0.038) | 5.27E-10 |  |
| 35 | PC ae C40:6 | Phosphatidylcholine acyl-alkyl C40:6 | -0.036 (-0.053, -0.018) | 7.50E-05 |  |
| 36 | PC ae C42:1 | Phosphatidylcholine acyl-alkyl C42:1 | -0.062 (-0.079, -0.044) | 1.76E-11 |  |
| 37 | PC ae C42:3 | Phosphatidylcholine acyl-alkyl C42:3 | -0.044 (-0.062, -0.026) | 1.00E-06 |  |
| 38 | PC ae C42:4 | Phosphatidylcholine acyl-alkyl C42:4 | -0.059 (-0.076, -0.041) | 7.55E-11 |  |
| 39 | PC ae C44:4 | Phosphatidylcholine acyl-alkyl C44:4 | -0.052 (-0.070, -0.034) | 1.02E-08 |  |
| 40 | PC ae C44:5 | Phosphatidylcholine acyl-alkyl C44:5 | -0.033 (-0.051, -0.016) | 2.06E-04 |  |
| 41 | PC ae C44:6 | Phosphatidylcholine acyl-alkyl C44:6 | -0.065 (-0.083, -0.047) | 9.90E-13 |  |
| 42 | SM (OH) C14:1 | Hydroxysphingomyeline C14:1 | -0.037 (-0.056, -0.019) | 8.40E-05 |  |
| 43 | SM C16:0 | Sphingomyeline C16:0 | -0.039 (-0.057, -0.021) | 2.30E-05 |  |
| 44 | SM C16:1 | Sphingomyeline C16:1 | -0.046 (-0.066, -0.07) | 3.00E-06 |  |
| 45 | SM (OH) C22:2 | Hydroxysphingomyeline C22:2 | -0.152 (-0.197, -0.103) | 2.44E-08 |  |
| 46 | SM C24:1 | Sphingomyeline C24:1 | -0.045 (-0.063, -0.028) | 3.27E-07 |  |
| 47 | H1 | Hexose | 0.042 (0.025, 0.060) | 3.00E-06 |  |

**d**

|  | Metabolites | | HOMA-IR | |
| --- | --- | --- | --- | --- |
| # | Abbreviation | Biochemical name | Beta (95% CI) | *P*-value |
| 1 | C14:2 | Tetradecadienylcarnitine | -0.081 (-0.123, -0.037) | 2.47E-04 |
| 2 | Ala | Alanine | 0.180 (0.134, 0.221) | 2.08E-15 |
| 3 | Arg | Arginine | 0.119 (0.075, 0.160) | 5.06E-08 |
| 4 | Glu | Glutamate | 0.091 (0.046, 0.134) | 6.40E-05 |
| 5 | Gly | Glycine | -0.081 (-0.124, -0.036) | 3.58E-04 |
| 6 | Ile | Isoleucine | 0.158 (0.109, 0.203) | 7.87E-11 |
| 7 | Leu | Leucine | 0.108 (0.060, 0.153) | 8.00E-06 |
| 8 | Phe | Phenylalanine | 0.100 (0.055, 0.143) | 1.30E-05 |
| 9 | Pro | Proline | 0.150 (0.104, 0.192) | 7.94E-11 |
| 10 | Tyr | Tyrosine | 0.181 (0.134, 0.222) | 5.14E-15 |
| 11 | Val | Valine | 0.177 (0.129, 0.221) | 1.45E-13 |
| 12 | Sarcosine | Sarcosine | 0.141 (0.094, 0.185) | 2.16E-09 |
| 13 | lysoPC a C17:0 | lysoPhosphatidylcholine acyl C17:0 | -0.091 (-0.132, -0.048) | 3.30E-05 |
| 14 | PC aa C30:0 | Phosphatidylcholine diacyl C30:0 | 0.081 (0.037, 0.122) | 2.34E-04 |
| 15 | PC aa C32:1 | Phosphatidylcholine diacyl C32:1 | 0.109 (0.063, 0.152) | 2.00E-06 |
| 16 | PC aa C34:1 | Phosphatidylcholine diacyl C34:1 | 0.085 (0.040, 0.127) | 1.94E-04 |
| 17 | PC aa C36:1 | Phosphatidylcholine diacyl C36:1 | 0.095 (0.051, 0.138) | 2.30E-05 |
| 18 | PC aa C40:1 | Phosphatidylcholine diacyl C40:1 | -0.082 (-0.124, -0.038) | 2.54E-04 |
| 19 | PC aa C42:0 | Phosphatidylcholine diacyl C42:0 | -0.082 (-0.125, -0.037) | 3.47E-04 |
| 20 | PC aa C42:4 | Phosphatidylcholine diacyl C42:4 | -0.091 (-0.132, -0.047) | 4.10E-05 |
| 21 | PC ae C34:3 | Phosphatidylcholine acyl-alkyl C34:3 | -0.111 (-0.153, -0.067) | 1.00E-06 |
| 22 | PC ae C40:1 | Phosphatidylcholine acyl-alkyl C40:1 | -0.088 (-0.130, -0.043) | 9.50E-05 |
| 23 | PC ae C40:3 | Phosphatidylcholine acyl-alkyl C40:3 | -0.096 (-0.139, -0.052) | 1.60E-05 |
| 24 | PC ae C42:0 | Phosphatidylcholine acyl-alkyl C42:0 | -0.104 (-0.146, -0.059) | 4.00E-06 |
| 25 | PC ae C42:1 | Phosphatidylcholine acyl-alkyl C42:1 | -0.123 (-0.166, -0.078) | 6.79E-08 |
| 26 | PC ae C42:3 | Phosphatidylcholine acyl-alkyl C42:3 | -0.081 (-0.123, -0.036) | 3.28E-04 |
| 27 | PC ae C42:4 | Phosphatidylcholine acyl-alkyl C42:4 | -0.100 (-0.142, -0.056) | 8.00E-06 |
| 28 | PC ae C44:4 | Phosphatidylcholine acyl-alkyl C44:4 | -0.098 (-0.140, -0.053) | 1.50E-05 |
| 29 | PC ae C44:6 | Phosphatidylcholine acyl-alkyl C44:6 | -0.083 (-0.126, -0.038) | 2.52E-04 |
| 30 | SM (OH) C14:1 | Hydroxysphingomyeline C14:1 | -0.088 (-0.133, -0.042) | 1.56E-04 |
| 31 | SM C16:0 | Sphingomyeline C16:0 | -0.097 (-0.140, -0.052) | 2.30E-05 |
| 32 | SM C16:1 | Sphingomyeline C16:1 | -0.131 (-0.177, -0.082) | 8.12E-08 |
| 33 | SM (OH) C16:1 | Hydroxysphingomyeline C16:1 | -0.088 (-0.132, -0.043) | 1.30E-04 |
| 34 | SM C18:1 | Sphingomyeline C18:1 | -0.100 (-0.146, -0.052) | 3.70E-05 |
| 35 | SM (OH) C22:2 | Hydroxysphingomyeline C22:2 | -0.152 (-0.197, -0.103) | 5.85E-10 |
| 36 | SM C24:1 | Sphingomyeline C24:1 | -0.088 (-0.130, -0.045) | 6.10E-05 |
| 37 | H1 | Hexose | 0.226 (0.180, 0.265) | 6.47E-24 |

**Supplementary Table S4**. Selection of significantly enriched metabolites in more than half of the PD and related conditions. 39 metabolites that results from both logistic and linear regression analyses showed significant concentration differences (*P* < 4.07E-04) corresponding to most of categories of PD (i. e., state of PD by ADA criteria, as well as fasting glucose or 2h-PPG, HbA1c and HOMA-IR levels).

|  | |  | | Logistic regression analysis | | | | Linear regression analysis | | | |
| --- | --- | --- | --- | --- | --- | --- | --- | --- | --- | --- | --- |
|  |  | PD Vs. NGT  (by the ADA criteria) | IFG Vs. NGT | IGT Vs. NGT | IFG+IGT Vs. NGT | PD Vs. NGT (by HbA1c) | PD Vs. NGT (by HOMA-IR) | Fasting glucose levels | 2h-PPG levels | HbA1c levels | HOMA-IR levels |
| # | Metabolites | OR (95% CI) | OR (95% CI) | OR (95% CI) | OR (95% CI) | OR (95% CI) | OR (95% CI) | Beta (95% CI) | Beta (95% CI) | Beta (95% CI) | Beta (95% CI) |
| 1 | Ala | 1.65 (1.47, 1.84) | 1.50 (1.28, 1.75) | 1.41 (1.21, 1.65) | 1.70 (1.42, 2.04) | 1.46 (1.30, 1.64) | 2.22 (1.83, 2.70) | 0.22 (0.18, 0.27) | 0.18 (0.13, 0.22) | 0.07 (0.06, 0.09) | 0.18 (0.13, 0.22) |
| 2 | Val | 1.69 (1.50, 1.91) | 1.37 (1.16, 1.61) | 1.69 (1.44, 1.99) | 1.51 (1.26, 1.81) | 1.63 (1.44, 1.84) | 1.83 (1.50, 2.22) | 0.19 (0.14, 0.24) | 0.22 (0.17, 0.27) | 0.09 (0.07, 0.11) | 0.18 (0.13, 0.22) |
| 3 | PC ae C34:3 | 0.63 (0.57, 0.71) | 0.68 (0.58, 0.79) | 0.64 (0.55, 0.75) | 0.53 (0.45, 0.63) | 0.74 (0.67, 0.83) | 0.56 (0.46, 0.67) | -0.19 (-0.24, -0.15) | -0.23 (-0.27, -0.18) | -0.05 (-0.07, -0.04) | -0.11 (-0.15, -0.07) |
| 4 | PC ae C40:3 | 0.73 (0.66, 0.81) | 0.73 (0.62, 0.85) | 0.72 (0.62, 0.84) | 0.71 (0.60, 0.84) | 0.79 (0.71, 0.88) | 0.70 (0.59, 0.83) | -0.12 (-0.16, -0.07) | -0.13 (-0.17, -0.08) | -0.05(-0.06, -0.03) | -0.10 (-0.14, -0.05) |
| 5 | PC ae C42:4 | 0.63 (0.57, 0.71) | 0.63 (0.54, 0.74) | 0.63 (0.54, 0.73) | 0.65 (0.55, 0.77) | 0.71 (0.64, 0.80) | 0.65 (0.55, 0.78) | -0.17 (-0.21, -0.12) | -0.16 (-0.20, -0.11) | -0.06 (-0.08, -0.04) | -0.10 (-0.14, -0.06) |
| 6 | PC ae C44:4 | 0.64 (0.57, 0.71) | 0.58 (0.50, 0.68) | 0.69 (0.59, 0.80) | 0.67 (0.58, 0.79) | 0.74 (0.67, 0.83) | 0.64 (0.53, 0.76) | -0.18 (-0.22, -0.14) | -0.12 (-0.16, -0.07) | -0.05 (-0.07, -0.03) | -0.10 (-0.14, -0.05) |
| 7 | PC ae C44:6 | 0.61 (0.55, 0.68) | 0.62 (0.53, 0.72) | 0.64 (0.55, 0.74) | 0.66 (0.56, 0.78) | 0.69 (0.62, 0.77) | 0.71 (0.60, 0.85) | -0.16 (-0.20, -0.11) | -0.13 (-0.18, -0.09) | -0.07 (-0.08, -0.05) | -0.08 (-0.13, -0.04) |
| 8 | SM C16:0 | 0.64 (0.58, 0.72) | 0.64 (0.55, 0.75) | 0.68 (0.58, 0.79) | 0.57 (0.48, 0.67) | 0.81 (0.72, 0.90) | 0.63 (0.52, 0.75) | -0.19 (-0.23, -0.14) | -0.18 (-0.23, -0.13) | -0.04 (-0.06, -0.02) | -0.10 (-0.14, -0.05) |
| 9 | SM C24:1 | 0.68 (0.61, 0.75) | 0.73 (0.63, 0.84) | 0.66 (0.57, 0.77) | 0.70 (0.60, 0.83) | 0.75 (0.67, 0.83) | 0.73 (0.62, 0.86) | -0.12 (-0.17, -0.08) | -0.15 (-0.19, -0.10) | -0.05 (-0.06, -0.03) | -0.09 (-0.13, -0.05) |
| 10 | Gly | 0.73 (0.65, 0.81) | 0.66 (0.55, 0.78) | 0.73 (0.62, 0.85) | 0.66 (0.55, 0.79) | - | 0.73 (0.61, 0.87) | -0.13 (-0.17, -0.08) | -0.14 (-0.19, -0.10) | -0.03 (-0.05, -0.02) | -0.08 (-0.12, -0.04) |
| 11 | PC aa C42:0 | 0.64 (0.57, 0.71) | 0.68 (0.59, 0.80) | 0.65 (0.55, 0.75) | 0.70 (0.59, 0.83) | 0.70 (0.62, 0.78) | - | -0.14 (-0.18, -0.09) | -0.13 (-0.18, -0.08) | -0.06 (-0.08, -0.04) | -0.08 (-0.13, -0.04) |
| 12 | PC ae C42:1 | 0.67 (0.60, 0.74) | - | 0.72 (0.62, 0.84) | 0.70 (0.59, 0.84) | 0.67 (0.60, 0.75) | 0.62 (0.52, 0.75) | -0.14 (-0.18, -0.09) | -0.18 (-0.22, -0.13) | -0.06 (-0.08, -0.04) | -0.12 (-0.17, -0.08) |
| 13 | SM (OH) C22:2 | 0.57 (0.50, 0.64) | - | 0.69 (0.58, 0.82) | 0.55 (0.45, 0.66) | 0.71 (0.63, 0.80) | 0.58 (0.48, 0.71) | -0.23 (-0.28, -0.18) | -0.14 (-0.19, -0.09) | -0.15 (-0.20, -0.10) | -0.15 (-0.20, -0.10) |
| 14 | SM C16:1 | 0.63 (0.56, 0.71) | 0.58 (0.49, 0.69) | - | 0.54 (0.45, 0.65) | 0.79 (0.70, 0.89) | 0.64 (0.53, 0.77) | -0.22 (-0.26, -0.17) | -0.15 (-0.20, -0.10) | -0.04 (-0.06, -0.07) | -0.13 (-0.18, -0.08) |
| 15 | PC ae C36:3 | 0.69 (0.62, 0.76) | 0.73 (0.63, 0.85) | 0.66 (0.57, 0.77) | 0.64 (0.54, 0.76) | 0.77 (0.69, 0.85) | 0.72 (0.60, 0.85) | -0.14 (-0.18, -0.10) | -0.19 (-0.24, -0.15) | -0.05 (-0.06, -0.03) | - |
| 16 | PC aa C42:1 | 0.67 (0.60, 0.74) | 0.69 (0.59, 0.80) | 0.72 (0.62, 0.84) | 0.68 (0.58, 0.81) | 0.73 (0.65, 0.81) | - | -0.13 (-0.18, -0.09) | -0.10 (-0.15, -0.06) | -0.06 (-0.08, -0.04) | - |
| 17 | PC ae C40:4 | 0.71 (0.64, 0.79) | 0.74 (0.64, 0.86) | 0.64 (0.55, 0.75) | - | 0.73 (0.65, 0.81) | 0.71 (0.60, 0.84) | -0.10 (-0.14, -0.06) | -0.14 (-0.18, -0.09) | -0.06 (-0.07, -0.04) | - |
| 18 | H1 | 1.74 (1.55, 1.95) | 3.09 (2.56, 3.74) | - | 3.25 (2.64, 3.99) | - | 2.5 (2.06, 3.07) | 0.50 (0.46, 0.54) | 0.13 (0.09, 0.18) | 0.04 (0.03, 0.06) | 0.23 (0.18, 0.27) |
| 19 | Glu | 1.43 (1.28, 1.59) | 1.34 (1.15, 1.56) | - | - | 1.28 (1.15, 1.43) | 1.41 (1.18, 1.69) | 0.11 (0.07, 0.16) | - | 0.05 (0.04, 0.07) | 0.09 (0.05, 0.13) |
| 20 | Pro | 1.34 (1.20, 1.50) | - | - | - | 1.26 (1.12, 1.40) | 1.69 (1.41, 2.03) | 0.14 (0.10, 0.19) | 0.10 (0.06, 0.15) | 0.04 (0.02, 0.06) | 0.15 (0.10, 0.19) |
| 21 | lysoPC a C18:2 | 0.70 (0.62, 0.78) | - | 0.67 (0.57, 0.78) | 0.64 (0.54, 0.76) | 0.72 (0.64, 0.80) | - | -0.10 (-0.15, -0.06) | -0.20 (-0.24, -0.15) | -0.05 (-0.07, -0.03) | - |
| 22 | PC ae C40:1 | 0.78 (0.69, 0.85) | - | 0.75 (0.64, 0.87) | - | 0.75 (0.68, 0.84) | - | -0.09 (-0.14, -0.05) | -0.13 (-0.18, -0.09) | -0.04 (-0.06, -0.02) | -0.09 (-0.13, -0.04) |
| 23 | SM (OH) C14:1 | 0.68 (0.61, 0.76) | 0.64 (0.54, 0.75) | - | 0.63 (0.53, 0.75) | - | - | -0.18 (-0.23, -0.13) | -0.10 (-0.15, -0.05) | -0.04 (-0.06, -0.02) | -0.09 (-0.13, -0.04) |
| 24 | Ile | - | - | 1.37 (1.16, 1.60) | - | 1.43 (1.27, 1.61) | 1.48 (1.22, 1.79) | - | 0.09 (0.04, 0.14) | 0.08 (0.06, 0.09) | 0.16 (0.11, 0.20) |
| 25 | Phe | - | - | 1.48 (1.27, 1.72) | - | 1.35 (1.20, 1.50) | - | 0.10 (0.05, 0.15) | 0.13 (0.08, 0.17) | 0.05 (0.03, 0.07) | 0.10 (0.06, 0.14) |
| 26 | Tyr | 1.27 (1.13, 1.41) | - | - | - | 1.29 (1.16, 1.45) | 1.55 (1.29, 1.87) | 0.13 (0.08, 0.17) | - | 0.05 (0.03, 0.06) | 0.18 (0.13, 0.22) |
| 27 | PC aa C36:1 | - | - | 1.36 (1.17, 1.57) | - | 1.30 (1.17, 1.45) | 1.41 (1.19, 1.68) | 0.08 (0.04, 0.13) | - | 0.05 (0.03, 0.07) | 0.10 (0.05, 0.14) |
| 28 | C18:1 | 1.41 (1.26, 1.57) | 1.55 (1.34, 1.80) | - | 1.38 (1.16, 1.63) | - | - | 0.14 (0.09, 0.18) | 0.10 (0.06, 0.15) | - | - |
| 29 | Arg | - | - | 1.42 (1.21, 1.66) | - | 1.24 (1.16, 1.38) | 1.67 (1.40, 1.99) | - | - | 0.05 (0.03, 0.06) | 0.12 (0.08, 0.16) |
| 30 | Met | 1.30 (1.16, 1.45) | - | 1.32 (1.14, 1.54) | - | 1.35 (1.20, 1.52) | - | - | 0.10 (0.05, 0.15) | 0.05 (0.04, 0.07) | - |
| 31 | Kynurenine | 0.79 (0.71, 0.88) | 0.65 (0.55, 0.76) | - | 0.64 (0.54, 0.77) | - | - | -0.11 (-0.15, -0.06) | -0.09 (-0.13, -0.04) | - | - |
| 32 | PC aa C34:1 | 1.32 (1.19, 1.47) | - | - | - | 1.24 (1.12, 1.39) | - | 0.09 (0.04, 0.13) | - | 0.04 (0.02, 0.06) | 0.09 (0.04, 0.13) |
| 33 | PC aa C40:5 | - | - | - | 1.42 (1.20, 1.69) | 1.24 (1.11, 1.38) | - | 0.12 (0.08, 0.17) | 0.12 (0.07, 0.16) | 0.05 (0.03, 0.06) | - |
| 34 | PC ae C30:0 | - | 0.67 (0.57, 0.78) | - | - | 0.81 (0.73, 0.91) | - | -0.13 (-0.17, -0.08) | -0.09 (-0.14, -0.05) | -0.04 (-0.06, -0.02) | - |
| 35 | PC ae C34:2 | 0.75 (0.68, 0.84) | - | 0.75 (0.64, 0.87) | 0.64 (0.54, 0.76) | - | - | -0.12 (-0.16, -0.07) | -0.15 (-0.20, -0.11) | - | - |
| 36 | PC ae C38:4 | 0.82 (0.74, 0.91) | - | 0.75 (0.65, 0.87) | - | 0.81 (0.73, 0.90) | - | - | -0.09 (-0.14, -0.05) | -0.03 (-0.05, -0.02) | - |
| 37 | PC ae C42:3 | 0.78 (0.70, 0.87) | - | 0.73 (0.63, 0.84) | - | 0.76 (0.68, 0.85) | - | - | -0.11 (-0.16, -0.07) | -0.04 (-0.06, -0.03) | - |
| 38 | SM (OH) C16:1 | 0.75 (0.67, 0.84) | 0.70 (0.60, 0.81) | - | - | - | 0.71 (0.59, 0.84) | -0.15 (-0.20, -0.10) | - | - | -0.09 (-0.13, -0.04) |
| 39 | SM C18:1 | 0.72 (0.64, 0.81) | 0.59 (0.50, 0.70) | - | 0.65 (0.54, 0.78) | - | - | -0.19 (-0.24, -0.14) | - | - | -0.10 (-0.15, -0.05) |

**Supplementary Table S5**. Spearman’s correlation coefficient between diabetic indices in 1,723 individuals

|  | Diabetic condition  (NGT/PD) | Fasting glucose (mg/dl) | 2-h glucose (mg/dl) | HbA1c (%) | HOMA-IR |
| --- | --- | --- | --- | --- | --- |
| Diabetic condition  (NGT/PD) | 1.000 | 0.778^*^ | 0.725^*^ | 0.631^*^ | 0.452 |
| Fasting glucose  (mg/dl) | 0.778^*^ | 1.000 | 0.529 | 0.435 | 0.541^*^ |
| 2-h glucose (mg/dl) | 0.725^*^ | 0.529 | 1.000 | 0.523 | 0.345 |
| HbA1c (%) | 0.631^*^ | 0.435 | 0.523 | 1.000 | 0.296 |
| HOMA-IR | 0.452 | 0.541^*^ | 0.345 | 0.296 | 1.000 |

* Correlation is significant at the 0.01 level (2-tailed).
